# Ocular safety signals of sphingosine-1-phosphate receptor modulators in postmarketing surveillance

**DOI:** 10.3389/fphar.2026.1908773

**Published:** 2026-09-25

**Authors:** Mei Fu, Jue Wang, Qingyi He, Nan Ma, Jie Zhang, Yangjun Li, Jing Han

**Affiliations:** Department of Ophthalmology, Tangdu Hospital, The Fourth Military Medical University, Xi’an, Shaanxi, China

**Keywords:** disproportionality analysis, FAERS, macular oedema, ocular adverse events, pharmacovigilance, postmarketing surveillance, reporting odds ratio, sphingosine-1-phosphate receptor modulators

## Abstract

**Background:**

Sphingosine-1-phosphate (S1P) receptor modulators are used for immune-mediated diseases, and macular oedema is an established ocular adverse effect. However, ocular-focused, class-wide postmarketing reporting across all five approved agents remains incompletely characterized.

**Methods:**

We analyzed U.S. Food and Drug Administration Adverse Event Reporting System (FAERS) data from 2004 Q1 through 2026 Q1 using report-level deduplication, approval-aligned windows, and primary-suspect exposure. Ocular events were assessed using a curated set of 52 Preferred Term (PT) strings. Sensitivity analyses included report-level macular/retinal oedema aggregation, same-indication active comparators, temporal and sparse-data analyses, restricted empirical-Bayes assessment, and external validation in Canada Vigilance. Exact-date time to onset was assessed for the aggregated phenotype.

**Results:**

The primary cohort included 98,843 primary-suspect reports, of which 8,812 (8.9%) were ocular, representing 12,088 unique ocular report–PT pairs. At the class level, 21 ocular PTs met primary signal criteria; macular oedema showed the strongest anatomically specific signal (n = 947; reporting odds ratio [ROR], 48.18; 95% confidence interval [CI], 44.84–51.76). Fingolimod had the broadest signal spectrum; macular oedema was the principal anatomically specific signal for siponimod and ozanimod. No ponesimod PT met primary criteria, and the etrasimod macular oedema estimate was sparse (n = 11). Of 36 primary drug–PT signals, 13 were High, 22 Moderate, and one Low. Report-level aggregation yielded a class-level ROR of 29.47 (95% CI, 27.62–31.45). In indication-restricted multiple sclerosis analyses, the association remained elevated versus all non-S1P disease-modifying therapies (ROR, 43.85; 95% CI, 35.75–53.78), while ulcerative colitis/inflammatory bowel disease analyses were sparse and exploratory. For fingolimod, the association persisted after exclusion of the first two post-approval years (ROR, 45.79; 95% CI, 37.26–56.26). Median time to onset was 93.0 days for fingolimod and 15.5 days for siponimod. Canada Vigilance showed a concordant class-level association (ROR, 74.00; 95% CI, 59.30–92.35).

**Conclusion:**

Macular/retinal oedema was the most consistent ocular reporting pattern in the S1P receptor modulator class. The association remained evident after phenotype aggregation and indication-restricted analysis and was also observed in Canada Vigilance. Sparse data for newer agents and residual confounding limit comparative interpretation.

## Introduction

Sphingosine-1-phosphate (S1P) receptor modulators are oral immunomodulatory agents used in immune-mediated diseases, particularly relapsing forms of multiple sclerosis (MS) and ulcerative colitis (UC) (McGinley and Cohen, 2021; Kitsou et al., 2024). By inducing functional antagonism of S1P receptors, these agents reduce lymphocyte egress from lymphoid tissues and limit inflammatory cell trafficking into target organs (McGinley and Cohen, 2021; Chun et al., 2021). Fingolimod was the first approved agent in this class, followed by more receptor-selective modulators, including siponimod, ozanimod, ponesimod, and etrasimod, which differ in receptor selectivity, clinical indications, and pharmacokinetic characteristics (Roy et al., 2021; Pérez-Jeldres et al., 2021). As S1P receptor modulators expand from MS to inflammatory bowel disease (IBD), real-world safety surveillance has become increasingly relevant (Sandborn et al., 2023).

Ocular adverse events are a recognized safety concern with S1P receptor modulators, and macular oedema is the most extensively characterized ophthalmic complication (Mandal et al., 2017). The FREEDOMS trial established the pivotal clinical-trial context for fingolimod-associated macular oedema, while pooled ophthalmic evaluations further characterized its occurrence and dose-related pattern (Kappos et al., 2010; Zarbin et al., 2013). Prior clinical and review evidence indicates that fingolimod-associated macular oedema often occurs within the first several months of treatment, and patients with diabetes mellitus or a history of uveitis may be at increased risk (Jain and Bhatti, 2012; Wang et al., 2022). Ocular adverse events have also been reported in clinical development programs for newer S1P receptor modulators, including siponimod, ozanimod, ponesimod, and etrasimod (Kappos et al., 2018; Comi et al., 2019; Kappos et al., 2021; Dubinsky et al., 2025). Reports of macular oedema associated with newer agents, including ponesimod and ozanimod, have also appeared in the case literature (Singh et al., 2024; Buckley et al., 2025; Mauceri et al., 2025). Beyond macular oedema, reported ocular findings include blurred vision, visual impairment, retinal vascular events, and subclinical changes in macular thickness, although these events are less consistently characterized (Mandal et al., 2017; Garcia-Martin et al., 2021; Nørgaard et al., 2020).

The mechanisms underlying S1P receptor modulator-associated ocular adverse events remain incompletely understood (Simon et al., 2021; Shiwani et al., 2021). S1P signaling is involved in vascular barrier regulation, endothelial function, immune-cell trafficking, and retinal homeostasis, suggesting that altered S1P receptor signaling may contribute to retinal vascular permeability or blood-retinal barrier dysfunction in susceptible individuals (Simon et al., 2021; Shiwani et al., 2021). Clinical trials are often underpowered for uncommon ocular events and generally focus on selected prespecified outcomes, underscoring the complementary role of postmarketing pharmacovigilance. Previous pharmacovigilance studies have evaluated broader adverse-event profiles of S1P receptor modulators or selected organ-system safety outcomes, while systematic reviews have summarized class-level safety and ocular adverse events associated with treatments for MS (Yang et al., 2024; Li et al., 2025; Lasa et al., 2021; Muñoz-Ortiz et al., 2021). However, ocular-focused, class-wide postmarketing reporting across all five approved S1P receptor modulators, including newer agents with relatively limited accumulated postmarketing experience, remains incompletely characterized.

In this study, we used the U.S. Food and Drug Administration (FDA) Adverse Event Reporting System (FAERS) to provide an ocular-focused, class-wide postmarketing characterization of reporting patterns associated with fingolimod, siponimod, ozanimod, ponesimod, and etrasimod. We aimed to characterize the spectrum and drug-specific distribution of ocular reporting patterns, with particular attention to anatomically specific posterior/retinal-macular findings and nonspecific visual symptoms that may be more susceptible to indication-related confounding. We further assessed the interpretability and robustness of these findings through related-term phenotype aggregation, same-indication active comparators, temporal and sparse-data sensitivity analyses, and targeted external validation in Canada Vigilance.

## Methods

### Study design and data sources

A retrospective pharmacovigilance analysis was conducted using FAERS, a publicly available spontaneous reporting database (Potter et al., 2025). Quarterly FAERS ASCII files from 2004 Q1 through 2026 Q1 were downloaded and processed. The study evaluated disproportional reporting of ocular adverse events associated with S1P receptor modulators. A targeted external validation was additionally conducted using Canada Vigilance data through 31 March 2026. Because the study used publicly available, de-identified spontaneous-reporting data and did not involve identifiable human participant data, institutional review board approval and informed consent were not required.

### Study drugs and report selection

The study drugs were five approved S1P receptor modulators: fingolimod, siponimod, ozanimod, ponesimod, and etrasimod. Exposure ascertainment used a structured dictionary of generic names, brand names, salt forms, and development codes, with matching limited to exact normalized or compact drug-name or active-ingredient aliases. The drug-alias dictionary for the five included agents and the FDA approval dates defining their analysis windows are provided in Supplementary Table S1.

Reports were included from each drug-specific FDA approval date through 2026 Q1. The analysis windows began on 21 September 2010 for fingolimod, 26 March 2019 for siponimod, 25 March 2020 for ozanimod, 18 March 2021 for ponesimod, and 12 October 2023 for etrasimod. Reports were retained if the FDA receipt date fell within the corresponding approval-aligned analysis window. The primary analysis was restricted to reports in which a target S1P receptor modulator was coded as the primary suspect drug to increase attribution specificity. Secondary-suspect, interacting, and concomitant drug roles were evaluated in sensitivity analyses. Pre-approval fingolimod/FTY720 records and investigational or exploratory S1P agents were excluded from the main analysis.

### Data processing and deduplication

FDA deleted-case identifiers were removed by CASEID before denominator construction. Duplicate reports and follow-up versions were resolved within each CASEID by retaining the highest available numeric CASEVERSION; ties or unavailable versions were resolved using FDA receipt date and report identifier. FDA receipt date determined approval-window eligibility, whereas event date and focal-drug treatment start date were used only for the time-to-onset (TTO) analysis (Potter et al., 2025; Banda et al., 2016). Drug-event pairs were counted at the unique report–Preferred Term (PT) level; repeated occurrences of the same PT within the same retained report were counted once. For class-level analyses, each retained report could contribute only once to a given PT, preventing duplicate class-level report–PT pairs when more than one included S1P receptor modulator was reported within the same report. Detailed identifier harmonization, date handling, and fallback rules are provided in the Supplementary Methods.

### Ocular adverse event identification and classification

Adverse events were analyzed at the Medical Dictionary for Regulatory Activities (MedDRA) PT level (Brown et al., 1999). The primary curated analysis set contained 52 source PT strings, a 34-term subset was used as a narrower sensitivity definition, and a 781-term broad exploratory set was reserved for supplementary analyses. Selected neuro-ophthalmic PTs that may reflect underlying MS, including optic neuritis and optic neuropathy, were excluded from the curated primary set and assessed in a separate exploratory neuro-ophthalmic analysis. Source PT strings used in the analyses were cross-referenced to current MedDRA version 29.0 for reproducibility. Complete term membership, current-version PT codes, and crosswalk details are provided in Supplementary Table S2 and the Supplementary Methods.

For clinical interpretation, ocular PTs were grouped into posterior/retinal-macular-choroidal disorders and nonspecific ocular/visual symptom reports. Posterior and retinal-macular terms included macular oedema, cystoid macular oedema, retinal oedema, maculopathy, retinal disorder, chorioretinopathy, and retinal haemorrhage. Nonspecific ocular or visual symptom terms included, among others, visual impairment, vision blurred, diplopia, visual acuity reduced, blindness, photophobia, scotoma, and eye disorder. These symptom-based terms were analyzed separately because of their lower clinical specificity. The presentation categories did not alter denominators, event definitions, or signal calculations.

### Disproportionality analysis

For each drug-PT analysis, the target set comprised deduplicated approval-window reports in which the focal S1P receptor modulator was coded as primary suspect. The comparator comprised all other eligible deduplicated FAERS reports in the same drug-specific window after exclusion of target reports. For class-level analyses, the target was the union of primary-suspect reports for the five included S1P receptor modulators, and the comparator window began on 21 September 2010, the earliest approval date in the class. In each 2 × 2 table, a represented target reports with the event, b target reports without the event, c comparator reports with the event, and d comparator reports without the event. Complete raw a/b/c/d cells are provided in Supplementary Table S3.

Reporting odds ratios (RORs) with 95% confidence intervals (CIs) were calculated for each drug-PT pair. ROR reflects relative reporting frequency (Rothman et al., 2004). Proportional reporting ratios (PRRs) and Pearson chi-square statistics were also calculated. A frequentist signal component required at least three target-event reports, PRR >2, and chi-square >4 (Evans et al., 2001). Information-component (IC) statistics were calculated as an additional disproportionality measure (Bate et al., 1998). IC025 was defined as the asymptotic lower 95% bound of the IC. A Haldane-Anscombe correction of 0.5 was applied to all 4 cells for ROR, PRR, IC, and IC025 calculations, whereas Pearson chi-square used the raw cells. A primary signal required a≥3, PRR>2, chi-square>4, IC025 > 0, and an ROR lower 95% confidence limit>1. Only tested drug-PT and class-PT pairs meeting all criteria were considered primary signals. Complete equations and implementation details are provided in the Supplementary Methods.

### Sensitivity and robustness analyses

Sensitivity analyses assessed the primary findings using the 34-PT sensitivity subset, an expanded exposure definition including primary or secondary suspect drug roles, a full-period comparator, and minimum target-event-count thresholds of n ≥ 5, n ≥ 10, and n ≥ 20. The broad exploratory PT analysis and the neuro-ophthalmic analysis were considered exploratory. Drug-PT robustness was evaluated across four analyses: the curated primary analysis, the 34-PT sensitivity analysis, primary-plus secondary-suspect sensitivity, and full-period-comparator sensitivity. High robustness required support in all four analyses and at least 10 target-event reports; signals involving ponesimod or etrasimod and selected visual-symptom PTs were not eligible for a High grade. Moderate robustness required support in at least three analyses and at least five target-event reports; remaining primary signals were classified as Low. The n ≥ 20 indicator identified sparse estimates. Detailed grading criteria and the class-level approach are provided in the Supplementary Methods and Supplementary Tables S4, S5.

A *post hoc* restricted empirical-Bayes sensitivity analysis using a two-component gamma-Poisson mixture model was applied to the broad exploratory drug-PT family to assess sparse estimates. The empirical Bayes geometric mean (EBGM) and corresponding posterior interval estimates are reported in Supplementary Table S6 (Dumouchel, 1999). Detailed model specification and estimation procedures are provided in the Supplementary Methods.

### Deduplicated report-level phenotype aggregation

To address co-reporting of related terms, macular oedema, cystoid macular oedema, and retinal oedema were grouped after CASEID-level deduplication as a report-level macular/retinal oedema phenotype, with each retained report counted once within the phenotype. The PT-level analysis remained the primary analysis. The phenotype definition, raw 2 × 2 cell counts, and corresponding class-level and drug-specific estimates are provided in Supplementary Table S7.

### Indication-restricted active-comparator and temporal sensitivity analyses

A same-indication active-comparator sensitivity evaluated the report-level macular/retinal oedema phenotype. Both target S1P and comparator exposures were restricted to primary-suspect reports with exact sequence-linked indication terms and drug-specific approval-aligned calendar windows. For MS, comparator pools included oral non-S1P therapies, platform injectables, high-efficacy therapies, anti-CD20 therapies, and all non-S1P disease-modifying therapies. Ozanimod and etrasimod were additionally evaluated against non-S1P UC/IBD comparator pools. Comparator definitions, co-exposure exclusions, feasibility thresholds, sparse-data rules, and detailed results are provided in Supplementary Table S8 and the Supplementary Methods.

A *post hoc* temporal sensitivity analysis used the exact-indication fingolimod versus all-non-S1P MS comparison to evaluate the first 2 years after approval, years 2–5, periods after years two and 5, and a calendar-quarter-stratified Cochran-Mantel-Haenszel common odds ratio. This analysis evaluated the influence of early post-approval reporting on the observed association.

### Canada Vigilance external validation

Targeted external validation was performed using the Canada Vigilance adverse-reaction extract through 31 March 2026. S1P exposure required the focal receptor modulator to be recorded as Suspect. Reports connected by explicit Duplicate links were collapsed into a single analysis unit, whereas records designated only Linked were retained separately. Drug-specific analyses used Canadian market-entry-aligned windows, and the class-level target comprised the union of unique eligible reports containing any included S1P receptor modulator as Suspect.

The primary external-validation endpoint was the report-level macular/retinal oedema phenotype used in the FAERS sensitivity analysis. A secondary targeted analysis separately evaluated the seven posterior/retinal-macular-choroidal PTs that met the class-level primary FAERS signal criteria. Each target population was compared with all other eligible Canada Vigilance reports in the corresponding market window. Detailed drug mapping, Canadian market-entry dates, report-link handling, sparse-data definitions, and multiplicity procedures are provided in the Supplementary Methods and Supplementary Table S9.

### Multiple-testing and sparse-data assessment

Pearson chi-square p values were adjusted using the Benjamini–Hochberg procedure within four analysis families: curated drug-PT, curated class-PT, broad drug-PT, and broad class-PT. Estimates with target-event counts <20 were classified as sparse. Primary signal classification followed the disproportionality criteria described above; adjusted q values provided a supplementary multiplicity assessment. Broad exploratory findings were treated as hypothesis-generating. (Supplementary Table S10 and Supplementary Data S1).

### TTO analysis

TTO was evaluated for the report-level macular/retinal oedema phenotype by linking the focal-drug treatment start date with the adverse-event onset date. Only reports with exact values for both dates contributed an interval; partial dates were not imputed. Zero-day intervals were retained, negative intervals were excluded, and no additional outlier exclusion was applied to valid nonnegative intervals. Valid-interval coverage was defined as the proportion of phenotype-positive reports with a valid nonnegative interval. Median and interquartile range (IQR) were reported only when valid-interval coverage was at least 30% and at least 10 valid nonnegative intervals were available (Supplementary Table S11).

### Statistical software

Python 3.9.6 was used for data processing, statistical analyses, and generation of tables and figures.

## Results

### Overall report characteristics

From 2004 Q1 through 2026 Q1, 21,881,120 raw FAERS DEMO rows were identified. After removal of 127,367 FDA deleted-case rows, 21,753,753 rows remained, and report-level deduplication yielded 18,450,566 unique reports. The final approval-date-aligned primary suspect S1P receptor modulator cohort included 98,843 class-level primary suspect reports, 8,812 ocular adverse event reports, and 12,088 unique ocular report–PT pairs, corresponding to an overall ocular reporting proportion of 8.9% (Figure 1). Among reports with available age fields, drug-level median age ranged from 44.0 to 55.0 years. Among reports with recorded sex, female was the most common category for each drug-specific cohort. Fingolimod accounted for most primary suspect reports (80,795; 81.7% of the S1P cohort), with 7,707 ocular adverse event reports and an ocular reporting proportion of 9.5%. Siponimod contributed 8,263 primary suspect reports (8.4%), 677 ocular adverse event reports, and an ocular reporting proportion of 8.2%. Ozanimod contributed 8,130 primary suspect reports (8.2%), 343 ocular adverse event reports, and an ocular reporting proportion of 4.2%. Ponesimod contributed 299 primary suspect reports (0.3%), 12 ocular adverse event reports, and an ocular reporting proportion of 4.0%. Etrasimod contributed 1,356 primary suspect reports (1.4%), 73 ocular adverse event reports, and an ocular reporting proportion of 5.4% (Table 1; Supplementary Figure S1). Leading recorded indications were MS or product used for unknown indication for fingolimod, siponimod, ozanimod, and ponesimod, whereas etrasimod reports were mainly coded as colitis ulcerative or colitis (Table 1).

**FIGURE 1:**
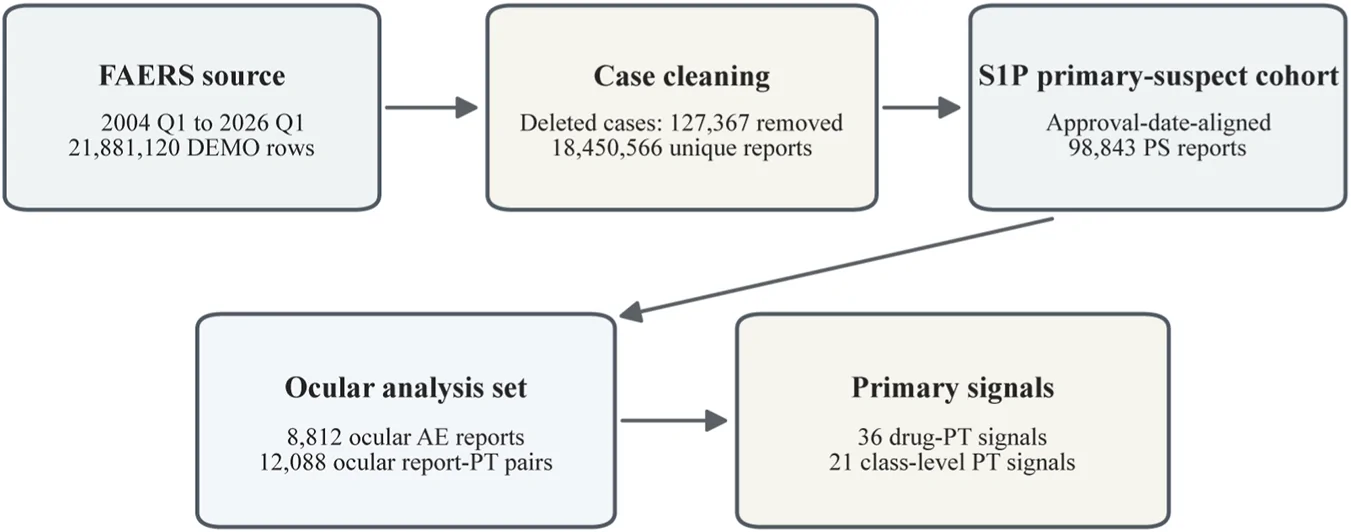
FAERS cohort construction. Flow diagram showing FAERS DEMO source records from 2004 Q1 through 2026 Q1, removal of FDA deleted cases, report-level deduplication, construction of the approval-date-aligned primary-suspect S1P receptor modulator cohort, and derivation of the ocular analysis set and primary signal outputs. Counts represent DEMO rows before report-level deduplication and reports thereafter, unless report–PT pairs or drug–PT signal pairs are specified. AE = adverse event; FAERS = FDA Adverse Event Reporting System; PS = primary suspect; PT = Preferred Term; S1P = sphingosine-1-phosphate.

**TABLE 1:** Cohort and reporting characteristics by S1P receptor modulator.

Drug	Reporting period	PS reports, n	Ocular AE reports, n	Ocular report-PT pairs, n	Ocular reporting proportion, %	Median age, y	Sex recorded/missing, n	Sex distribution among recorded reports, n	Leading indications
Fingolimod	2010–09-21 to 2026 Q1	80,795	7,707	10,712	9.5	44.0	76,752/4,043	F (59,493); M (16,867); NS (293); UNK (99)	Multiple sclerosis (47,764); product used for unknown indication (27,186); relapsing-remitting multiple sclerosis (9,580)
Siponimod	2019–03-26 to 2026 Q1	8,263	677	885	8.2	55.0	7,718/545	F (5807); M (1911)	Product used for unknown indication (3479); multiple sclerosis (3336); secondary progressive multiple sclerosis (2149)
Ozanimod	2020–03-25 to 2026 Q1	8,130	343	383	4.2	49.0	7,740/390	F (5498); M (2238); UNK (4)	Multiple sclerosis (4445); product used for unknown indication (2568); colitis ulcerative (1846)
Ponesimod	2021–03-18 to 2026 Q1	299	12	13	4.0	48.0	237/62	F (188); M (48); UNK (1)	Product used for unknown indication (145); multiple sclerosis (138); relapsing-remitting multiple sclerosis (16)
Etrasimod	2023–10-12 to 2026 Q1	1,356	73	95	5.4	46.0	1,229/127	F (662); M (567)	Colitis ulcerative (519); colitis (37); proctitis ulcerative (16)

AE, adverse event; F, female; M, male; NS, not specified; PS, primary suspect; PT, preferred term; S1P, sphingosine-1-phosphate; UNK, unknown. Counts are FAERS, reports unless report-Preferred Term pairs are specified. Sex distribution is calculated among reports with recorded sex; sex missing is shown separately.

### Class-level ocular reporting signals

At the class level, 21 ocular PTs met the primary signal criteria, including seven posterior/retinal-macular-choroidal PTs and 14 nonspecific ocular/visual symptom PTs. The most frequently reported ocular PTs were visual impairment (n = 2,868), vision blurred (n = 2,784), macular oedema (n = 947), eye pain (n = 908), diplopia (n = 626), visual acuity reduced (n = 529), eye disorder (n = 508), and blindness (n = 474) (Figure 2; Supplementary Table S4). The strongest class-level disproportionality estimate was observed for macular oedema (n = 947; ROR, 48.18; 95% CI, 44.84–51.76), representing the most prominent anatomically specific posterior/retinal-macular signal. Other posterior/retinal-macular-choroidal signals included retinal oedema (n = 76; ROR, 9.18; 95% CI, 7.30–11.55), cystoid macular oedema (n = 72; ROR, 7.86; 95% CI, 6.21–9.94), retinal disorder (n = 57; ROR, 5.14; 95% CI, 3.95–6.68), maculopathy (n = 65; ROR, 4.00; 95% CI, 3.13–5.11), chorioretinopathy, and retinal haemorrhage. Scotoma (n = 15; ROR, 9.86; 95% CI, 5.92–16.44) was categorized as a nonspecific symptom-based report and interpreted cautiously. Other nonspecific ocular/visual symptom reports included diplopia, visual impairment, vision blurred, blindness, eye disorder, and photopsia; these were interpreted separately from anatomically specific posterior segment signals (Figure 2; Supplementary Table S4).

**FIGURE 2:**
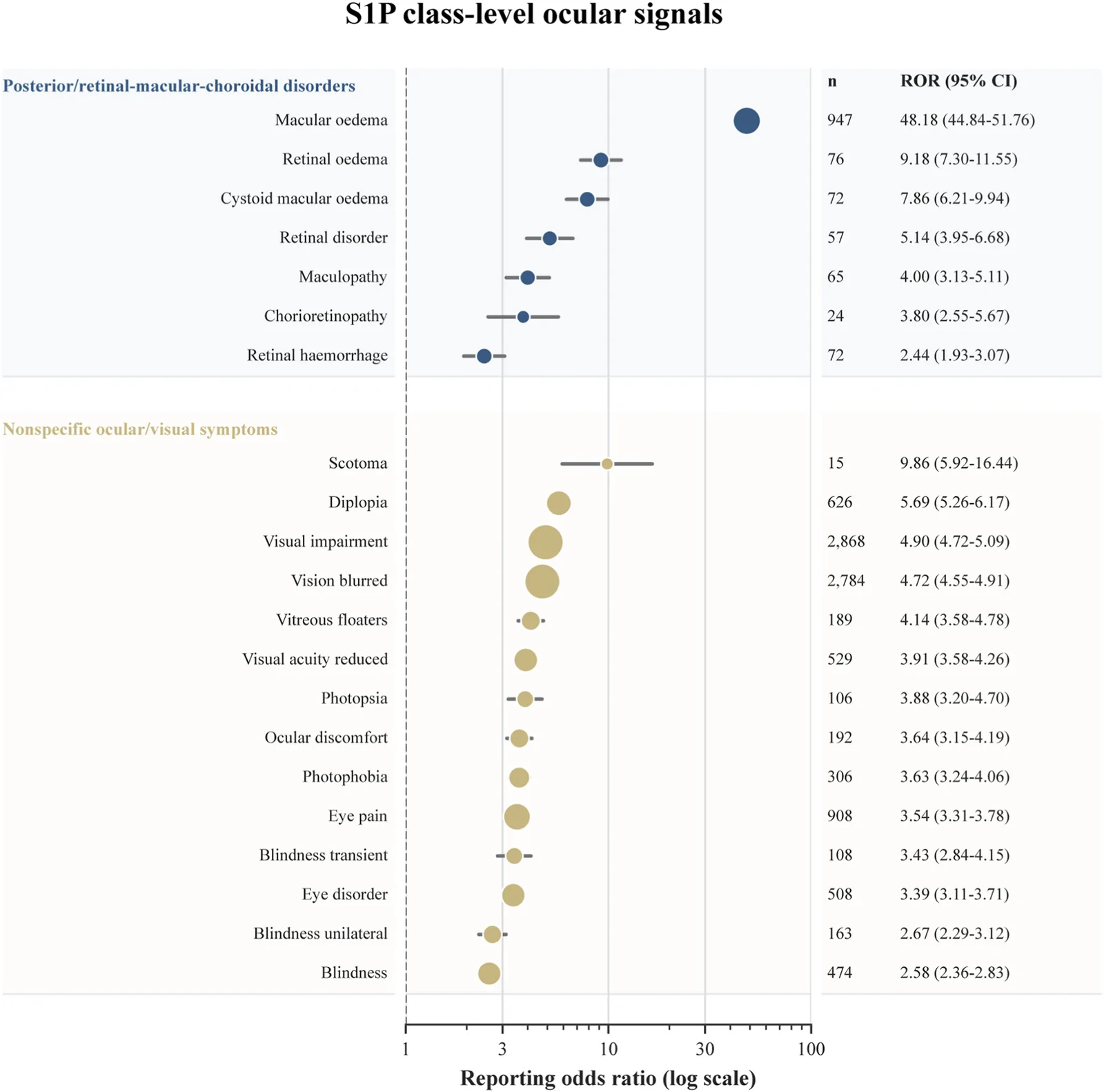
S1P class-level ocular reporting signals. Forest plot of class-level ocular PTs meeting the primary signal criteria for S1P receptor modulators. Points indicate reporting odds ratios, horizontal bars indicate 95% confidence intervals, and point size reflects report count. PTs are grouped as posterior/retinal-macular-choroidal disorders or nonspecific ocular/visual symptom reports. The x-axis is shown on a logarithmic scale.

### Drug-specific ocular reporting signals

Drug-specific ocular reporting signals are summarized in Figures 3, 4 and Supplementary Table S12. The distribution of ocular report–PT pairs by presentation category is shown in Supplementary Figure S2.

**FIGURE 3:**
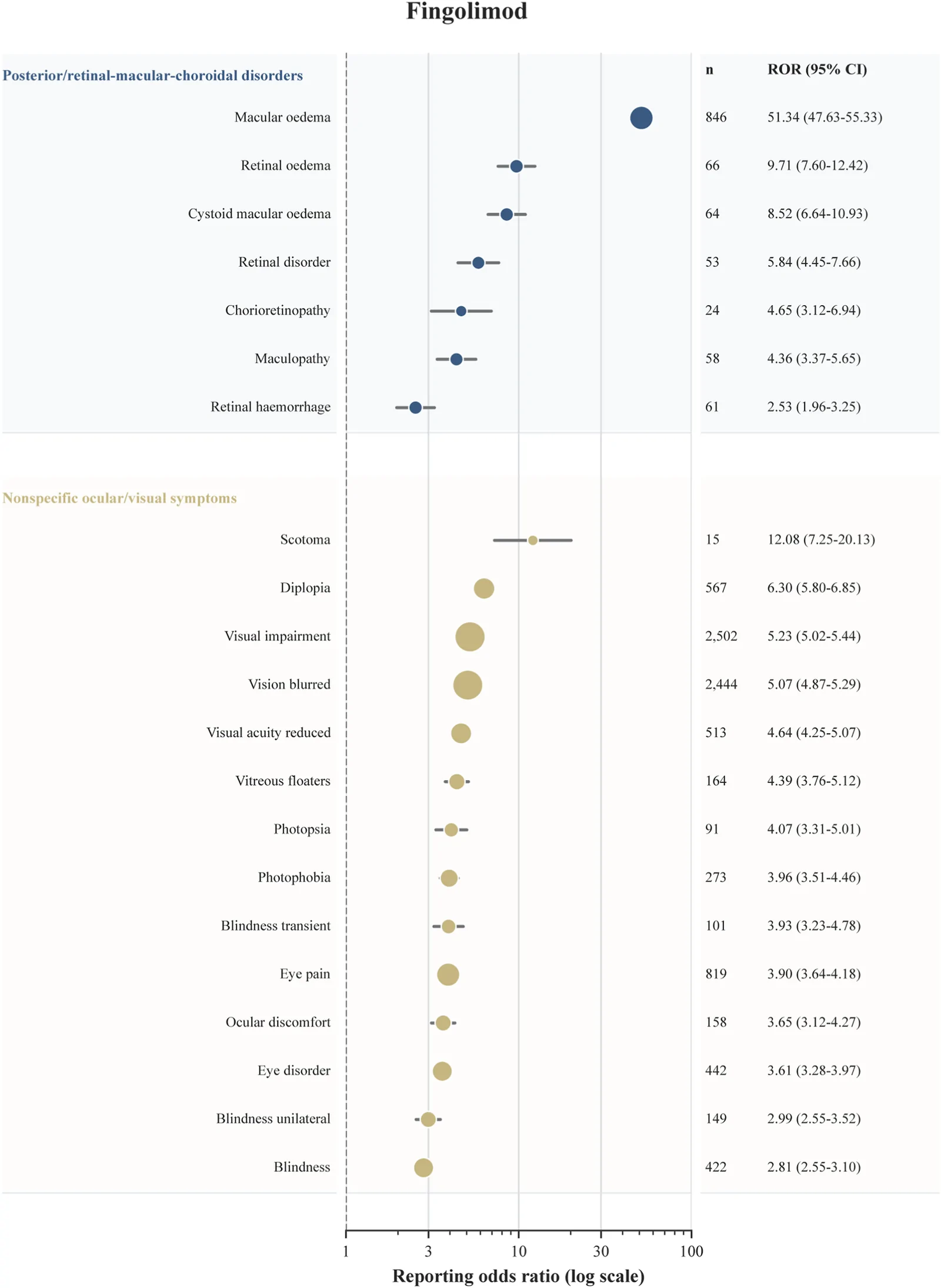
Fingolimod ocular reporting signals. Forest plot of fingolimod-specific ocular PTs meeting the primary signal criteria. Points indicate reporting odds ratios, horizontal bars indicate 95% confidence intervals, and point size reflects report count. PTs are grouped as posterior/retinal-macular-choroidal disorders or nonspecific ocular/visual symptom reports. The x-axis is shown on a logarithmic scale.

**FIGURE 4:**
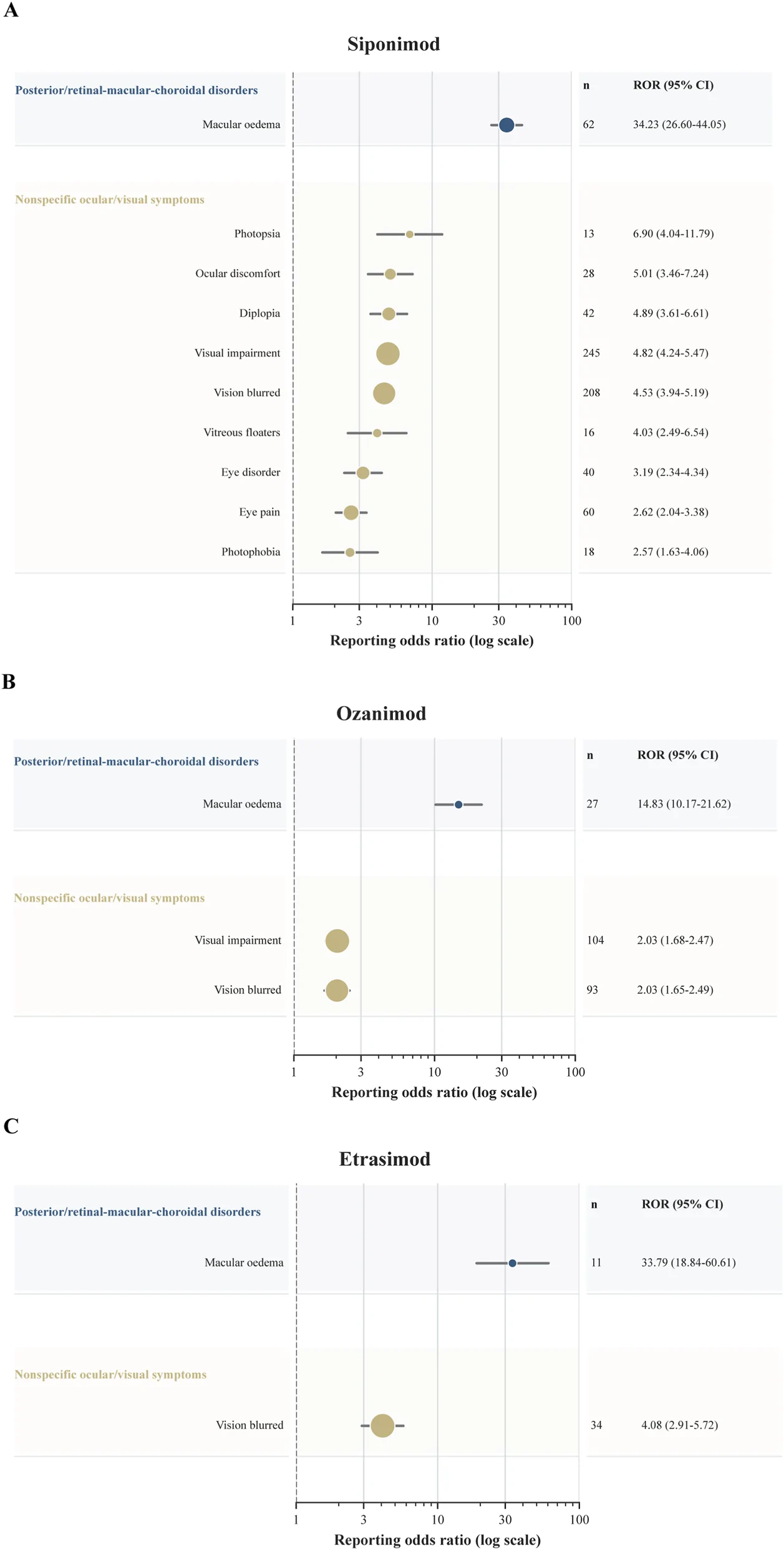
Ocular reporting signals for siponimod, ozanimod, and etrasimod. Agent-specific forest plots of ocular PTs meeting the primary signal criteria for siponimod **(A)**, ozanimod **(B)**, and etrasimod **(C)**. Points indicate reporting odds ratios, horizontal bars indicate 95% confidence intervals, and point size reflects report count. PTs are grouped as posterior/retinal-macular-choroidal disorders or nonspecific ocular/visual symptom reports. The x-axis is shown on a logarithmic scale. Ponesimod is not shown because no ocular PT met the primary signal criteria in the approval-date-aligned primary-suspect analysis.

### Fingolimod

Fingolimod contributed 80,795 primary suspect reports and 7,707 ocular adverse event reports, with an ocular reporting proportion of 9.5%. The most frequently reported ocular PTs were visual impairment (n = 2,502), vision blurred (n = 2,444), macular oedema (n = 846), eye pain (n = 819), diplopia (n = 567), and visual acuity reduced (n = 513). The highest ROR estimates were observed for macular oedema (n = 846; ROR, 51.34; 95% CI, 47.63–55.33), scotoma (n = 15; ROR, 12.08; 95% CI, 7.25–20.13), retinal oedema (n = 66; ROR, 9.71; 95% CI, 7.60–12.42), cystoid macular oedema (n = 64; ROR, 8.52; 95% CI, 6.64–10.93), and diplopia (n = 567; ROR, 6.30; 95% CI, 5.80–6.85) (Figure 3; Supplementary Table S12). Fingolimod had seven posterior/retinal-macular-choroidal signals, including macular oedema, retinal oedema, cystoid macular oedema, retinal haemorrhage, maculopathy, retinal disorder, and chorioretinopathy. Frequent nonspecific ocular/visual symptom reports included visual impairment, vision blurred, eye pain, diplopia, visual acuity reduced, eye disorder, blindness, and photophobia (Figure 3; Supplementary Table S12).

### Siponimod

Siponimod contributed 8,263 primary suspect reports and 677 ocular adverse event reports, with an ocular reporting proportion of 8.2%. The most frequently reported ocular PTs were visual impairment (n = 245), vision blurred (n = 208), macular oedema (n = 62), eye pain (n = 60), diplopia (n = 42), and eye disorder (n = 40). The highest ROR estimates were observed for macular oedema (n = 62; ROR, 34.23; 95% CI, 26.60–44.05), photopsia (n = 13; ROR, 6.90; 95% CI, 4.04–11.79), ocular discomfort (n = 28; ROR, 5.01; 95% CI, 3.46–7.24), diplopia (n = 42; ROR, 4.89; 95% CI, 3.61–6.61), and visual impairment (n = 245; ROR, 4.82; 95% CI, 4.24–5.47). Macular oedema was the only posterior/retinal-macular-choroidal signal for siponimod; remaining primary signals were nonspecific ocular/visual symptom reports (Figure 4A; Supplementary Table S12).

### Ozanimod

Ozanimod contributed 8,130 primary suspect reports and 343 ocular adverse event reports, with an ocular reporting proportion of 4.2%. Three drug-specific ocular PTs met primary signal criteria: visual impairment (n = 104), vision blurred (n = 93), and macular oedema (n = 27). Macular oedema was the only posterior/retinal-macular-choroidal signal and had the highest ROR estimate (n = 27; ROR, 14.83; 95% CI, 10.17–21.62). Visual impairment (n = 104; ROR, 2.03; 95% CI, 1.68–2.47) and vision blurred (n = 93; ROR, 2.03; 95% CI, 1.65–2.49) were classified as nonspecific visual symptom reports (Figure 4B; Supplementary Table S12).

### Ponesimod

Ponesimod contributed 299 primary suspect reports and 12 ocular adverse event reports, with an ocular reporting proportion of 4.0%. No ocular PT met the primary signal criteria. In the restricted empirical-Bayes sensitivity, none of the selected ponesimod ocular terms had a lower 5th posterior percentile >1 (Supplementary Table S6).

### Etrasimod

Etrasimod contributed 1,356 primary suspect reports and 73 ocular adverse event reports, with an ocular reporting proportion of 5.4%. Two ocular PTs met primary signal criteria: macular oedema and vision blurred. Macular oedema had the stronger disproportionality signal (n = 11; ROR, 33.79; 95% CI, 18.84–60.61; IC025, 0.18), followed by vision blurred (n = 34; ROR, 4.08; 95% CI, 2.91–5.72) (Figure 4C; Supplementary Table S12). The macular oedema estimate was sparse (n = 11). In the restricted empirical-Bayes sensitivity analysis, the corresponding EBGM was 22.43, with 5th and 95th posterior percentiles of 13.43 and 35.66, respectively (Supplementary Table S6).

### Sensitivity and robustness analyses

Sensitivity and robustness analyses generally supported the primary signal pattern. Among the 36 primary drug-PT signals, 13 were graded High, 22 Moderate, and one Low; ozanimod–vision blurred was the only Low signal (Figure 5; Supplementary Table S5). The 34-PT sensitivity subset retained 27 drug-specific and 16 class-level signals. The full-period comparator retained 35 drug-specific and 21 class-level signals, while the expanded primary- or secondary-suspect analysis retained 36 drug-specific and 21 class-level signals. In exploratory analyses, 81 drug-specific and 68 class-level pairs met the signal criteria in the broad exploratory set (Supplementary Table S10), while 12 drug-specific pairs met the criteria in the neuro-ophthalmic analysis.

**FIGURE 5:**
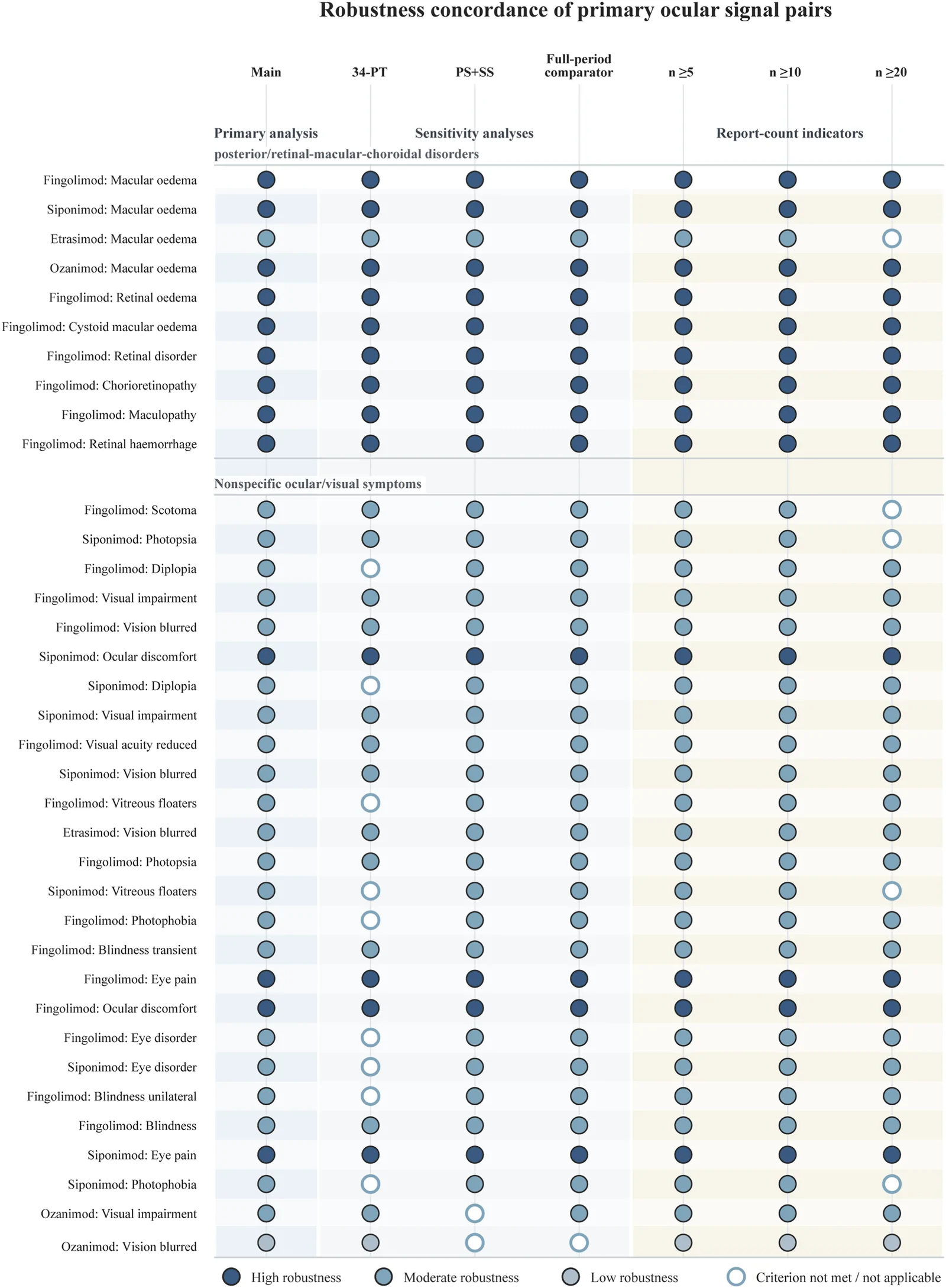
Robustness concordance matrix of primary ocular signal pairs. The matrix summarizes 36 primary drug–PT signal pairs across the primary and sensitivity analyses. Main denotes the approval-date-aligned primary-suspect analysis; 34-PT denotes the 34-PT sensitivity analysis; PS + SS denotes inclusion of primary- or secondary-suspect drug roles; and Full-period comparator denotes use of the full FAERS comparator period. The n ≥ 5 and n ≥ 10 columns show the report-count requirements used for Moderate and High robustness, respectively; the n ≥ 20 column indicates whether the non-sparse report-count threshold was met. Filled circles indicate that the corresponding analysis or count criterion was met. Open circles indicate that the criterion was not met or, in the 34-PT column, that the PT was not included in the 34-PT subset. Estimates with n < 20 were considered sparse. Marker color indicates High, Moderate, or Low robustness.

### Report-level phenotype aggregation

When macular oedema, cystoid macular oedema, and retinal oedema were aggregated at the report level, the class-level phenotype included 1,066 target reports and 6,735 comparator reports (ROR, 29.47; 95% CI, 27.62–31.45; PRR, 29.16; IC025, 4.22). Drug-specific estimates were elevated for fingolimod (n = 950; ROR, 31.65; 95% CI, 29.56–33.88), siponimod (n = 71; ROR, 21.86; 95% CI, 17.28–27.64), and ozanimod (n = 32; ROR, 9.74; 95% CI, 6.89–13.77), whereas ponesimod (n = 2) and etrasimod (n = 11) remained sparse (Supplementary Table S7).

### Indication-restricted active-comparator and temporal sensitivity analyses

In the indication-restricted MS analyses, class-level estimates remained elevated across the non-S1P comparator pools. Against all non-S1P MS disease-modifying therapies, 693 of 67,185 S1P reports and 106 of 447,862 comparator reports contained the macular/retinal oedema phenotype (ROR, 43.85; 95% CI, 35.75–53.78). Ponesimod comparisons did not meet the minimum event-count requirement. In the UC/IBD analyses, the class-level comparison against all non-S1P UC/IBD therapies included 22 phenotype-positive reports among 2,293 S1P reports and 10 among 76,959 comparator reports (ROR, 72.59; 95% CI, 34.85–151.19); all UC/IBD comparisons remained sparse and were considered exploratory (Supplementary Table S8).

For fingolimod, exclusion of the first 2 years after approval yielded an ROR of 45.79 (95% CI, 37.26–56.26), and the calendar-quarter-stratified common odds ratio was 44.01 (95% CI, 35.20–55.04) (Supplementary Table S8).

### Multiple-testing and sparse-data assessment

After Benjamini–Hochberg adjustment, 61 of 260 curated drug-PT tests and 29 of 52 curated class-PT tests had q ≤ 0.05; 218 and 23 estimates, respectively, were based on fewer than 20 target-event reports. In the broad exploratory analyses, 190 of 3,905 drug-PT tests and 131 of 781 class-PT tests had q ≤ 0.05; 3,827 and 716 estimates, respectively, were sparse. Broad exploratory findings were treated as hypothesis-generating (Supplementary Table S10; Supplementary Data S1).

### TTO analysis

The criteria for reporting median TTO were met for fingolimod and siponimod. For fingolimod, 322 of 950 phenotype-positive reports had valid exact-date nonnegative intervals (33.9%), with a median TTO of 93.0 days (IQR, 20.2–238.8). For siponimod, 22 of 71 reports had valid intervals (31.0%), with a median TTO of 15.5 days (IQR, 3.0–73.5). Ozanimod, ponesimod, and etrasimod did not satisfy both criteria required for reporting a median; feasibility results are provided in Supplementary Table S11.

### Canada Vigilance external validation

In Canada Vigilance, 109 of 4,683 class-level S1P Suspect reports contained the macular/retinal oedema phenotype, compared with 292 of 904,557 comparator reports (ROR, 74.00; 95% CI, 59.30–92.35; IC025, 3.74). Fingolimod had 93 phenotype-positive reports among 3,661 Suspect reports (ROR, 76.89; 95% CI, 60.86–97.13), whereas siponimod had 16 among 693 reports and remained sparse (ROR, 61.61; 95% CI, 36.95–102.72).

Ozanimod had one phenotype-positive report among 108 Suspect reports; no eligible ponesimod Suspect report was identified, and etrasimod had no phenotype-positive report among 238 Suspect reports. Among the seven targeted posterior/retinal-macular-choroidal PTs, macular oedema was the only non-sparse class-level estimate (n = 104; ROR, 121.04; 95% CI, 94.72–154.68); the remaining PTs were based on 1–9 target reports (Supplementary Table S9; Supplementary Figure S3).

## Discussion

In this FAERS pharmacovigilance analysis, we provide an ocular-focused, class-wide characterization of postmarketing reporting patterns across all five approved S1P receptor modulators, including newer agents with relatively limited accumulated postmarketing experience. At the class level, the strongest and most biologically plausible findings centered on macular/retinal oedema-related reports. Macular oedema was the most prominent individual PT, with 947 reports and a high disproportionality estimate, while retinal oedema and cystoid macular oedema also met primary signal criteria. Drug-specific analyses showed that fingolimod contributed the largest number of ocular reports and the broadest spectrum of signals. Siponimod and ozanimod showed more limited posterior segment signal patterns, with macular oedema as the principal anatomically specific signal. Etrasimod showed limited but retained signals for macular oedema and vision blurred, and ponesimod did not meet primary signal criteria under the current analysis.

The macular oedema signal identified in this study is consistent with the established ophthalmic safety profile of S1P receptor modulators. The FREEDOMS trial established the pivotal clinical-trial context for fingolimod-associated macular oedema, and pooled ophthalmic evaluations subsequently reported confirmed macular oedema in 0.3% of patients receiving the approved 0.5-mg dose and 1.2% receiving the 1.25-mg dose (Kappos et al., 2010; Zarbin et al., 2013). Prior reviews and case-based evidence also indicate that fingolimod-associated macular edema often occurs within the first several months of treatment and may improve after drug discontinuation or ophthalmic treatment (Jain and Bhatti, 2012; Wang et al., 2022). Clinical-development data also provide context for newer S1P receptor modulators, including siponimod in EXPAND and ozanimod in SUNBEAM (Kappos et al., 2018; Comi et al., 2019). OPTIMUM reported macular oedema in six ponesimod-treated participants and none receiving teriflunomide, and a subsequent case report described ponesimod-associated macular oedema (Kappos et al., 2021; Singh et al., 2024). Recent case reports have also described ozanimod-associated macular oedema, including an asymptomatic optical coherence tomography (OCT)-detected case (Buckley et al., 2025; Mauceri et al., 2025). Integrated long-term ozanimod safety data and the etrasimod development program indicate that macular oedema remained uncommon in clinical-development populations (Dubinsky et al., 2025; Rubin et al., 2026). Prior systematic reviews have also examined the broader safety of S1P receptor modulators and ocular adverse events associated with pharmacologic treatment in MS (Lasa et al., 2021; Muñoz-Ortiz et al., 2021). Together, these findings support macular oedema as the most consistently documented ocular safety concern across the class.

The drug-specific pattern observed in this study should be interpreted in the context of differential market exposure, receptor selectivity, indication, and reporting behavior. Fingolimod accounted for most ocular reports and showed primary signals for macular oedema, retinal oedema, cystoid macular oedema, maculopathy, retinal disorder, chorioretinopathy, and retinal haemorrhage. This broad profile is plausible because fingolimod was the earliest approved agent, has the longest postmarketing exposure, and has a well-recognized ophthalmic monitoring requirement that may increase detection and reporting (Mandal et al., 2017; Zarbin et al., 2013). The absence of a primary ponesimod signal should not be interpreted as evidence of lower ocular risk, given the small number of ocular reports and the documented occurrence of macular oedema in the OPTIMUM trial and a subsequent case report (Kappos et al., 2021; Singh et al., 2024). Similarly, the etrasimod findings should be viewed as preliminary because of limited report volume and short postmarketing exposure. Although its macular oedema signal remained supported in the empirical-Bayes sensitivity, it was based on only 11 reports and is insufficient for comparative safety conclusions.

In indication-restricted analyses, the class-level macular/retinal oedema association remained elevated against non-S1P MS comparators, although the UC/IBD analyses were limited by sparse events. For fingolimod, the association also persisted after exclusion of the first two post-approval years and after calendar-quarter stratification, showing that the association persisted beyond the early post-approval period. A concordant class-level association was also observed in Canada Vigilance, providing cross-database support for the principal macular/retinal oedema finding. Drug-specific support beyond fingolimod was limited by sparse or absent eligible reports.

The predominance of macular and retinal fluid-related events is biologically plausible. S1P signaling participates in endothelial barrier regulation, vascular permeability, immune-cell trafficking, and retinal homeostasis (Simon et al., 2021; Shiwani et al., 2021). Mechanistic studies suggest that S1P receptor 1 signaling supports endothelial barrier integrity through G-protein–mediated cytoskeletal stabilization and junctional organization, whereas disruption of this signaling may weaken barrier function (Anwar and Mehta, 2020). Retina-specific evidence also suggests that pericyte-derived S1P contributes to retinal endothelial barrier properties by modulating adhesion proteins and endothelial stability (McGuire et al., 2011). In addition, S1P receptors are expressed in retinal and subretinal tissues, and S1P signaling has been implicated in neovascular and permeability-related retinal disease (Alshaikh et al., 2022). These data provide a plausible mechanistic framework through which functional antagonism of S1P receptors could contribute to macular oedema or retinal fluid accumulation in susceptible patients.

Several non-macular signals require more cautious interpretation. Visual impairment, vision blurred, diplopia, photopsia, scotoma, and blindness were frequently reported, particularly with fingolimod and siponimod, but these events are clinically nonspecific and prone to confounding. MS itself can involve visual and neuro-ophthalmic manifestations, including optic neuritis, visual field abnormalities, reduced contrast sensitivity, and retinal structural changes, which may overlap with nonspecific visual symptom reports in FAERS (Gelfand et al., 2012; Balcer, 2006). Therefore, although these visual symptom signals are important for clinical awareness, they should not be interpreted as drug-specific ocular toxicities without additional clinical confirmation. For etrasimod, confounding differs because the treated population is largely composed of patients with UC. Ocular extraintestinal manifestations, including episcleritis, scleritis, and uveitis, are recognized in inflammatory bowel disease (Gros and Kaplan, 2023; Rogler et al., 2021).

Posterior segment signals beyond macular oedema also merit attention. Fingolimod showed primary signals for retinal haemorrhage, maculopathy, retinal disorder, and chorioretinopathy. These findings align with prior reviews noting that retinal hemorrhage and retinal vascular occlusion have been described in association with fingolimod, although the evidence is much more limited than for macular edema (Mandal et al., 2017). A published case report described macular haemorrhage after fingolimod therapy, with OCT-confirmed macular thickening and visual improvement after discontinuation; disruption of the inner blood-retinal barrier was proposed as a possible mechanism (Bhatti et al., 2013). These non-edematous posterior segment findings should be considered hypothesis-generating, given possible confounding by underlying neurologic or vascular disease and reporting bias.

Exact-date TTO estimates for the macular/retinal oedema phenotype were available for fingolimod and siponimod. The median TTO was 93.0 days for fingolimod and 15.5 days for siponimod. The fingolimod estimate is broadly consistent with previous evidence that fingolimod-associated macular oedema often occurs within the first several months of treatment (Zarbin et al., 2013; Jain and Bhatti, 2012; Wang et al., 2022). Given the limited availability of exact onset dates and the influence of treatment duration and reporting practices in spontaneous-report systems, these estimates should be interpreted descriptively and should not be used to define monitoring intervals (Karimi et al., 2013).

Clinically, these findings reinforce continued ophthalmic monitoring for patients receiving S1P receptor modulators. Monitoring should follow agent-specific prescribing information and be guided by individual ocular risk and clinical findings. Patients with diabetes mellitus, prior uveitis, pre-existing macular or retinal disease, or new visual symptoms may warrant closer ophthalmic attention (Mandal et al., 2017; Zarbin et al., 2013; Jain and Bhatti, 2012; Wang et al., 2022). New visual symptoms should prompt evaluation for macular oedema and other posterior segment abnormalities.

This study has limitations inherent to spontaneous-report pharmacovigilance. First, FAERS is subject to underreporting, stimulated reporting, missing clinical information, variable report quality, and potential residual duplication despite deduplication (Potter et al., 2025). Second, FAERS lacks denominator data on exposed patients, precluding estimation of incidence, prevalence, or absolute risk and limiting causal inference (Potter et al., 2025). Third, disproportionality estimates may be unstable for low-count events and newer agents, particularly ponesimod and etrasimod; findings from the broad exploratory PT set should therefore be considered hypothesis-generating. Fourth, direct comparisons across agents remain limited by differences in approval dates, market uptake, indication, disease severity, treatment channeling, ophthalmic monitoring, label awareness, and reporting behavior. The indication-restricted and temporal analyses provided additional context but cannot eliminate residual confounding or reporting bias. Fifth, although selected neuro-ophthalmic PTs were excluded from the primary analysis and nonspecific visual symptom reports were interpreted separately, residual confounding by indication remains possible because MS and UC can themselves cause ocular or visual manifestations that overlap with the events studied (Gelfand et al., 2012; Balcer, 2006; Rogler et al., 2021). A formal systematic review was not performed. FAERS reports also generally lack detailed clinical narratives, OCT or fundus imaging, visual acuity, and rechallenge information required for case-level adjudication and phenotype confirmation. Finally, Canada Vigilance is also a spontaneous-report system and shares many of the same reporting limitations as FAERS.

In conclusion, macular/retinal oedema was the most consistent ocular reporting pattern in the S1P receptor modulator class, while other posterior segment and nonspecific visual signals varied across agents. The association remained evident after report-level phenotype aggregation and in indication-restricted MS analyses, and was also observed at the class level in Canada Vigilance. These findings extend the postmarketing characterization of ocular reporting across all five approved S1P receptor modulators, while sparse data for newer agents and residual confounding limit comparative interpretation.

## Data Availability

The original contributions presented in the study are included in the article/Supplementary Material, further inquiries can be directed to the corresponding author.

## Supplementary material

SUPPLEMENTARY FIGURE S1 Ocular reporting volume and ocular reporting proportion by drug. Panel **(A)** shows ocular adverse event report counts for each S1P receptor modulator. Panel **(B)** shows ocular reporting proportions, calculated as the number of ocular adverse event reports divided by the number of primary-suspect reports for each drug.

SUPPLEMENTARY FIGURE S2 Composition of ocular report–PT pairs by presentation category. Stacked bars show the distribution of ocular report–PT pairs across posterior/retinal-macular-choroidal disorders and nonspecific ocular/visual symptom reports for each S1P receptor modulator. Percentages are based on ocular report–PT pairs within each drug, not on all primary-suspect reports.

SUPPLEMENTARY FIGURE S3 Canada Vigilance external validation of the report-level macular/retinal oedema phenotype. Points show reporting odds ratios and horizontal lines show 95% confidence intervals for the S1P class and individual agents in Canadian market-entry-aligned Suspect-report cohorts. Filled markers indicate estimates based on ≥20 phenotype-positive target reports, and open markers indicate estimates based on <20 phenotype-positive target reports. Ponesimod had no eligible Suspect reports, and no phenotype-positive Suspect reports were observed for etrasimod.
