# Supplementary material for: Ocular safety signals of sphingosine-1-phosphate receptor modulators in postmarketing surveillance
Source: Front Pharmacol. 2026 Sep 25;17:1908773. doi: 10.3389/fphar.2026.1908773 (PMC13649686; doi:10.3389/fphar.2026.1908773)
Supplement: Supplementary file 1 [file Supplementaryfile1.docx]

**Supplementary Methods**

The following sections provide additional methodological detail for analyses of sphingosine-1-phosphate (S1P) receptor modulators in the U.S. Food and Drug Administration (FDA) Adverse Event Reporting System (FAERS) and for the Canada Vigilance external validation.

**FAERS data source and analysis period**

Quarterly public FAERS files from 2004 Q1 through 2026 Q1 were used. Drug-specific analyses were restricted to FDA approval-date-aligned windows through 2026 Q1. The analysis windows began on September 21, 2010 for fingolimod, March 26, 2019 for siponimod, March 25, 2020 for ozanimod, March 18, 2021 for ponesimod, and October 12, 2023 for etrasimod. Exact FDA_DT was used when available. Reports with missing or unparseable FDA_DT were eligible for quarter-level fallback based on the corresponding approval quarter; however, no report in the primary S1P receptor modulator cohorts required this fallback. The class-level window began on September 21, 2010, the earliest approval date among the five included agents.

**Deleted reports and deduplication**

FDA deleted-case identifiers were removed by CASEID before denominator construction. Legacy and modern identifiers were harmonized as PRIMARYID/ISR for report ID and CASEID/CASE for case ID. Within each CASEID, the report with the highest numeric CASEVERSION was retained. If versions tied or were unavailable, FDA_DT and then report ID were used to select the final record. Of 21,881,120 raw DEMO rows, 127,367 deleted-case rows were removed, leaving 21,753,753 rows before report-level deduplication and 18,450,566 retained reports. FDA_DT determined analysis-window eligibility. EVENT_DT and focal-drug THER START_DT were used only for the time-to-onset (TTO) analysis.

**Drug exposure definition**

The primary analysis was restricted to reports in which fingolimod, siponimod, ozanimod, ponesimod, or etrasimod was recorded as the primary suspect drug. Drug matching used exact normalized DRUGNAME or PROD_AI values and compact exact matching for development codes. Drug-specific generic names, brand names, salt forms, and development codes were included. Supplementary Table S1 lists the drug-alias dictionary for the five included agents and the FDA approval dates defining their analysis windows.

**Ocular Preferred Term definitions and MedDRA crosswalk**

Ocular adverse events were identified from FAERS REAC event text at the Medical Dictionary for Regulatory Activities (MedDRA) Preferred Term (PT) level. The analysis used the 52-source-term curated primary set, 34-source-term sensitivity subset, and 781-term broad exploratory set defined in the main Methods. For reproducibility, each source string was cross-referenced to MedDRA version 29.0 using exact normalized PT-text matching. When no exact PT match was found, an exact Lowest Level Term (LLT) match was mapped to its current parent PT. Because public FAERS REAC files do not provide report-level historical MedDRA codes or versions, the source PT strings were mapped to MedDRA version 29.0 to provide a consistent current-version crosswalk. The 52 curated and 34 sensitivity source strings mapped to 51 and 33 unique current-version parent PT codes, respectively. Supplementary Table S2 provides complete term membership and the MedDRA crosswalk.

**Clinical presentation categories**

For reporting and clinical interpretation, PTs were grouped as posterior/retinal-macular-choroidal disorders or nonspecific ocular/visual symptom reports, consistent with the main manuscript. The nonspecific group included, among others, visual impairment, vision blurred, diplopia, scotoma, photopsia, blindness, visual acuity reduced, and eye disorder. These symptom-based terms were interpreted separately because they are less anatomically specific and may overlap with visual and neuro-ophthalmic manifestations of multiple sclerosis (MS). These presentation categories did not affect denominators, event definitions, or primary signal criteria.

**2 × 2 contingency tables and signal criteria**

For each drug-PT analysis, the target set comprised deduplicated reports within the drug-specific approval window in which the focal S1P receptor modulator was coded as primary suspect (PS). The comparator comprised all other eligible deduplicated FAERS reports in the same drug-specific window after exclusion of target reports. For the class-level analysis, the target was the union of PS reports for the five S1P receptor modulators, and the comparator used the class window beginning September 21, 2010.

In each 2 × 2 table, a represented target reports with the event, b target reports without the event, c comparator reports with the event, and d comparator reports without the event. The raw a/b/c/d counts are reported in Supplementary Table S3. A Haldane-Anscombe correction of 0.5 was applied to all four cells for the reporting odds ratio (ROR), proportional reporting ratio (PRR), and information component (IC) calculations; Pearson chi-square was calculated from the raw cells. ROR was calculated as (a+0.5)(d+0.5)/[(b+0.5)(c+0.5)], with its 95% confidence interval (CI) calculated on the log scale. PRR was [(a+0.5)/(a+b+1)]/[(c+0.5)/(c+d+1)]. Pearson chi-square was N(ad-bc)^2/[(a+b)(c+d)(a+c)(b+d)], where N=a+b+c+d.

For IC, E=[(a+b+1)(a+c+1)]/(a+b+c+d+2), IC=log2[(a+0.5)/E], and the standard error of the IC [SE(IC)]=sqrt[1/(a+0.5)+1/E]/ln(2). IC025, defined as the asymptotic lower 95% bound of the IC, was calculated as IC-1.96 SE(IC). A primary signal required a≥3, PRR>2, chi-square>4, IC025>0, and an ROR lower 95% confidence limit>1.

**Restricted empirical-Bayes sensitivity**

A post hoc restricted empirical-Bayes sensitivity analysis using a two-component gamma-Poisson model was applied to the 3,905 broad exploratory drug-PT cells to assess sparse estimates. For each cell, the observed count was O=a, and the expected count under independence was E=(a+b)(a+c)/(a+b+c+d). Of the 3,905 cells, 3,472 had E>0 and were included in model fitting. Maximum-likelihood estimation used 24 starting values; 20 converged to solutions within 0.01 negative log-likelihood units of the optimum, and the best-fitting solution was not on a parameter boundary. The empirical Bayes geometric mean (EBGM) was the exponentiated posterior expected log relative reporting ratio, and EB05 and EB95 were the posterior 5th and 95th percentiles. The model was fitted within the broad exploratory drug-PT family and was therefore treated as a restricted empirical-Bayes sensitivity analysis. Supplementary Table S6 reports results for selected low-report drug-PT pairs.

**Robustness grading**

Drug-PT robustness was assessed across four analyses: the curated primary analysis, the 34-PT sensitivity analysis, primary- plus secondary-suspect (PS+SS) sensitivity, and full-period-comparator sensitivity. Among primary signals, High robustness required support in all four analyses and at least 10 target-event reports; Moderate robustness required support in at least three analyses and at least five reports; remaining primary signals were classified as Low. For PTs not included in the 34-PT sensitivity subset, that analysis was recorded as not applicable and contributed no support to the robustness count.

Signals involving ponesimod or etrasimod, or the following visual-symptom PTs, were not eligible for a High grade: scotoma, diplopia, visual impairment, vision blurred, visual acuity reduced, photopsia, blindness transient, eye disorder, blindness unilateral, and blindness. These restrictions applied to the High grade; Moderate and Low grades followed the support and report-count criteria above. Presentation categories were used for clinical interpretation. The n≥20 indicator identified sparse estimates. Supplementary Table S5 and Figure 5 show the 36 primary drug-PT signals and their robustness grades.

Class-level robustness was evaluated separately. High robustness required support in the 34-PT sensitivity, PS+SS, and full-period-comparator analyses with at least 10 target-event reports. Moderate robustness required PS+SS and full-period-comparator support with at least five target-event reports; remaining class-level primary signals were classified as Low. High robustness was not assigned to the following visual-symptom PTs: scotoma, diplopia, visual impairment, vision blurred, visual acuity reduced, photopsia, blindness transient, eye disorder, blindness unilateral, and blindness. When these PTs otherwise met the High criteria, they were classified as Moderate. Supplementary Table S4 reports the class-level assignments.

**Deduplicated report-level phenotype aggregation**

After CASEID-level deduplication, macular oedema, cystoid macular oedema, and retinal oedema were combined into a report-level macular/retinal oedema phenotype. Each retained report was counted once within the phenotype, regardless of how many of the included PTs were reported in that case. Target and comparator cohorts followed the corresponding class-level or drug-specific definitions and approval-aligned windows used in the PT-level analyses. Phenotype-level ROR, PRR, and IC025 were calculated from the resulting report-level 2 × 2 tables using the same 0.5 Haldane-Anscombe correction as in the PT-level analyses. The PT-level analysis remained the primary analysis. Supplementary Table S7 provides the phenotype definition, a/b/c/d cell counts, and corresponding class-level and drug-specific estimates.

**Indication-restricted active-comparator and temporal sensitivity analyses**

A same-indication active-comparator sensitivity analysis evaluated the report-level macular/retinal oedema phenotype. Both target S1P and comparator exposures were restricted to primary-suspect reports identified using exact normalized or compact drug aliases, drug-specific approval-aligned windows, and exact sequence-linked indication terms. For MS, comparator pools comprised oral non-S1P disease-modifying therapies, platform injectables, high-efficacy therapies, anti-CD20 therapies, and all non-S1P MS disease-modifying therapies. For ulcerative colitis/inflammatory bowel disease (UC/IBD), comparator pools comprised biologic therapies, Janus kinase (JAK) inhibitors, and all non-S1P UC/IBD therapies.

Target reports containing another S1P primary-suspect drug or a comparator primary-suspect drug were excluded. Comparator reports containing an S1P primary-suspect drug or more than one distinct comparator primary-suspect drug were also excluded. Minimum analysis criteria were at least 100 target reports, 300 comparator reports, three target phenotype reports, and three comparator phenotype reports. Comparisons with either phenotype count below 20 were classified as sparse. Non-sparse MS comparisons were considered the principal active-comparator sensitivity analyses, whereas sparse MS estimates were interpreted as supportive. UC/IBD comparisons were considered exploratory because event counts were sparse. Ponesimod comparisons that did not meet the minimum event-count criterion were reported as feasibility-only. Supplementary Table S8 provides the comparator-pool definitions and detailed active-comparator and temporal sensitivity results.

A post hoc temporal sensitivity analysis was performed for the fingolimod versus all non-S1P MS comparison using the same exact-indication definition and exact FDA receipt dates. Separate 2 × 2 estimates were calculated for the first two years after approval, years 2–5, the period after year 2, and the period after year 5. A Cochran-Mantel-Haenszel common odds ratio was also calculated across calendar-quarter strata. This analysis evaluated whether the fingolimod association was concentrated in the early post-approval period.

**Time-to-onset analysis**

TTO was evaluated for the report-level macular/retinal oedema phenotype by linking focal-drug THER START_DT with DEMO EVENT_DT. Only reports with exact values for both dates contributed an interval; partial dates were not imputed. Zero-day intervals were retained, negative intervals were excluded and counted, and no additional outlier exclusion was applied to valid nonnegative intervals. Valid-interval coverage was calculated as the proportion of phenotype-positive reports with a valid nonnegative interval. Median and interquartile range were reported only when valid-interval coverage was at least 30% and at least 10 valid nonnegative intervals were available. Supplementary Table S11 reports phenotype-positive report counts, interval availability, excluded negative intervals, zero-day intervals, and drug-specific TTO summaries.

**Multiple-testing and sparse-data assessment**

Pearson chi-square p values were adjusted using the Benjamini-Hochberg (BH) procedure separately within four analysis families: curated drug-PT, curated class-PT, broad exploratory drug-PT, and broad exploratory class-PT. Estimates with target-event counts <20 were classified as sparse. Primary signal classification followed the disproportionality criteria described above; BH-adjusted q values were used as a supplementary multiplicity assessment. Broad exploratory findings were treated as hypothesis-generating. Supplementary Table S10 summarizes the four test families, and Supplementary Data S1 contains the complete row-level multiple-testing and sparse-data results.

**Canada Vigilance external validation**

External validation used the Canada Vigilance adverse reaction extract through March 31, 2026. The Reports file contained 1,252,950 current report rows, with one row per REPORT_ID and REPORT_NO; VERSION_NO identified the current report version. DATINTRECEIVED (Initial Received Date) determined market-window eligibility, and three reports with a missing Initial Received Date were excluded. Reports connected by explicit Duplicate links were collapsed into connected components, while records designated only Linked remained separate, yielding 1,190,984 analysis units. A sensitivity analysis without Duplicate-link collapse yielded 110 phenotype-positive reports among 4,824 target reports and an ROR of 70.47 (95% CI, 56.66–87.65), compared with 109 phenotype-positive reports among 4,683 target reports and an ROR of 74.00 (95% CI, 59.30–92.35) after Duplicate-link collapse.

S1P receptor modulator exposure was identified using DRUG_PRODUCT_ID links from the Canada Vigilance product and ingredient files together with exact normalized generic and brand names in the Report Drug file. Exposure required DRUGINVOLV_ENG to equal Suspect. No matching ponesimod or Ponvory product or Report Drug entry was identified in the extract; ponesimod therefore had no eligible target reports and was non-estimable.

The primary external-validation endpoint was the report-level macular/retinal oedema phenotype defined by the exact PT_NAME_ENG values Macular oedema, Cystoid macular oedema, and Retinal oedema, with each report counted once within the phenotype after Duplicate-link collapse. A secondary PT-level analysis separately evaluated the seven posterior/retinal-macular-choroidal PTs that met the class-level primary FAERS signal criteria: Macular oedema, Retinal oedema, Cystoid macular oedema, Retinal disorder, Maculopathy, Chorioretinopathy, and Retinal haemorrhage. These PTs were analyzed separately, and no broader ocular signal screening was performed in Canada Vigilance. All target reaction rows in the extract were coded using MedDRA version 29.0.

Drug-specific windows extended from Canadian market entry through March 31, 2026: fingolimod, March 28, 2011; siponimod, April 24, 2020; ozanimod, November 12, 2020; ponesimod, November 1, 2021; and etrasimod, April 18, 2024. The class-level window began on March 28, 2011. At class level, the target was the union of unique eligible reports containing any included S1P receptor modulator as Suspect; reports containing more than one included modulator were counted once. For each analysis, a and b represented eligible target reports with and without the endpoint, and c and d represented all other eligible Canada Vigilance reports in the same market window with and without the endpoint. The same 0.5 correction used in the FAERS analysis was applied to ROR, PRR, and IC calculations.

Estimates with fewer than 20 endpoint-positive target reports were classified as sparse, and those with fewer than three were considered feasibility-only. The secondary PT analysis comprised 42 planned comparisons (seven PTs across six analysis levels). Thirty-five comparisons had evaluable Pearson p values; the seven ponesimod comparisons were non-estimable. BH adjustment used a family size of 42, with q values calculated for the 35 evaluable comparisons. Supplementary Table S9 provides the complete Canada Vigilance estimates.
