# Supplementary material for: Ocular safety signals of sphingosine-1-phosphate receptor modulators in postmarketing surveillance
Source: Front Pharmacol. 2026 Sep 25;17:1908773. doi: 10.3389/fphar.2026.1908773 (PMC13649686; doi:10.3389/fphar.2026.1908773)
Supplement: Supplementary file 2 [file Table1.docx]

**Supplementary Table S1. Drug aliases and FDA approval-date analysis windows.**

**Panel A. FDA approval dates used to define drug-specific FAERS analysis windows**

| **Generic name** | **Brand name** | **FDA approval date (analysis start)** |
| --- | --- | --- |
| Fingolimod | Gilenya | 2010-09-21 |
| Siponimod | Mayzent | 2019-03-26 |
| Ozanimod | Zeposia | 2020-03-25 |
| Ponesimod | Ponvory | 2021-03-18 |
| Etrasimod | Velsipity | 2023-10-12 |

**Panel B. Drug aliases used for FAERS exposure identification**

| **Drug** | **Drug name or alias** | **Alias type** |
| --- | --- | --- |
| Fingolimod | fingolimod | Generic name |
| Fingolimod | fingolimod hydrochloride | Salt form |
| Fingolimod | fingolimod hcl | Salt form |
| Fingolimod | gilenya | Brand name |
| Fingolimod | tascenso | Brand name |
| Fingolimod | tascenso odt | Brand name |
| Fingolimod | fty720 | Development code |
| Fingolimod | fty-720 | Development code |
| Siponimod | siponimod | Generic name |
| Siponimod | mayzent | Brand name |
| Siponimod | baf312 | Development code |
| Siponimod | baf-312 | Development code |
| Ozanimod | ozanimod | Generic name |
| Ozanimod | ozanimod hydrochloride | Salt form |
| Ozanimod | zeposia | Brand name |
| Ozanimod | rpc1063 | Development code |
| Ozanimod | rpc-1063 | Development code |
| Ponesimod | ponesimod | Generic name |
| Ponesimod | ponvory | Brand name |
| Ponesimod | act128800 | Development code |
| Ponesimod | act-128800 | Development code |
| Etrasimod | etrasimod | Generic name |
| Etrasimod | etrasimod arginine | Salt form |
| Etrasimod | velsipity | Brand name |
| Etrasimod | apd334 | Development code |
| Etrasimod | apd-334 | Development code |

S1P, sphingosine-1-phosphate; FDA, U.S. Food and Drug Administration; Q, quarter. All FAERS analysis windows extended through 2026 Q1. Drug matching used exact normalized DRUGNAME or PROD_AI values and compact exact matching for development codes. Panel B lists the generic names, brand names, salt forms, and development codes used to identify the five included S1P receptor modulators.
