# Supplementary material for: Ocular safety signals of sphingosine-1-phosphate receptor modulators in postmarketing surveillance
Source: Front Pharmacol. 2026 Sep 25;17:1908773. doi: 10.3389/fphar.2026.1908773 (PMC13649686; doi:10.3389/fphar.2026.1908773)
Supplement: Supplementary file 3 [file Table10.docx]

**Supplementary Table S10. Multiple-testing and sparse-data assessment.**

| **Test family** | **Tests, n** | **Rows meeting signal** **criteria, n** | **Rows with target-event** **n < 20, n** | **Rows with BH q <= 0.05, n** |
| --- | --- | --- | --- | --- |
| Curated drug-PT family | 260 | 36 | 218 | 61 |
| Curated class-PT family | 52 | 21 | 23 | 29 |
| Broad exploratory drug-PT family | 3,905 | 81 | 3,827 | 190 |
| Broad exploratory class-PT family | 781 | 68 | 716 | 131 |

BH, Benjamini-Hochberg; q value, BH-adjusted p value; PT, Preferred Term. Benjamini-Hochberg adjustment was performed separately within the curated drug-PT, curated class-PT, broad exploratory drug-PT, and broad exploratory class-PT families. Estimates with fewer than 20 target-event reports were classified as sparse. BH-adjusted q values were used as a supplementary multiplicity assessment and did not define signal classification. Broad exploratory findings were treated as hypothesis-generating. Complete row-level multiple-testing and sparse-data results are provided in Supplementary Data S1.
