# Supplementary material for: Ocular safety signals of sphingosine-1-phosphate receptor modulators in postmarketing surveillance
Source: Front Pharmacol. 2026 Sep 25;17:1908773. doi: 10.3389/fphar.2026.1908773 (PMC13649686; doi:10.3389/fphar.2026.1908773)
Supplement: Supplementary file 4 [file Table11.docx]

**Supplementary Table S11. Time-to-onset analysis for the report-level macular/retinal oedema phenotype.**

| **Drug** | **Phenotype-positive reports, n** | **Valid nonnegative onset intervals, n** | **Valid-interval coverage, %** | **Reports without both exact dates, n** | **Zero-day intervals, n** | **Negative intervals excluded, n** | **TTO summary** | **Median onset, days** | **IQR, days** |
| --- | --- | --- | --- | --- | --- | --- | --- | --- | --- |
| Fingolimod | 950 | 322 | 33.9 | 626 | 39 | 2 | Median/IQR reported | 93.0 | 20.2-238.8 |
| Siponimod | 71 | 22 | 31.0 | 48 | 3 | 1 | Median/IQR reported | 15.5 | 3.0-73.5 |
| Ozanimod | 32 | 4 | 12.5 | 27 | 0 | 1 | Feasibility only | NA | NA |
| Ponesimod | 2 | 1 | 50.0 | 1 | 0 | 0 | Feasibility only | NA | NA |
| Etrasimod | 11 | 1 | 9.1 | 10 | 1 | 0 | Feasibility only | NA | NA |

TTO, time to onset; IQR, interquartile range; NA, not applicable. TTO was calculated only when exact EVENT_DT and exact focal-drug THER START_DT were available. Partial dates were not imputed. Zero-day intervals were retained, negative intervals were excluded, and no additional outlier exclusion was applied to valid nonnegative intervals. Valid-interval coverage was calculated as the proportion of phenotype-positive reports with a valid nonnegative interval. Reports without both exact dates were calculated as phenotype-positive reports minus valid nonnegative intervals and excluded negative intervals. Medians and IQRs were reported only when valid-interval coverage was >=30% and at least 10 valid nonnegative intervals were available.
