# Supplementary material for: Ocular safety signals of sphingosine-1-phosphate receptor modulators in postmarketing surveillance
Source: Front Pharmacol. 2026 Sep 25;17:1908773. doi: 10.3389/fphar.2026.1908773 (PMC13649686; doi:10.3389/fphar.2026.1908773)
Supplement: Supplementary file 5 [file Table12.docx]

**Supplementary Table S12. Drug-specific primary ocular reporting signals.**

| **Drug** | **Preferred Term** | **Presentation group** | **Reports, n** | **ROR (95% CI)** | **PRR** | **IC025** | **Robustness** |
| --- | --- | --- | --- | --- | --- | --- | --- |
| Fingolimod | Macular oedema | Posterior / retinal-macular-choroidal disorders | 846 | 51.34 (47.63-55.33) | 50.81 | 4.75 | High |
| Siponimod | Macular oedema | Posterior / retinal-macular-choroidal disorders | 62 | 34.23 (26.60-44.05) | 33.98 | 2.96 | High |
| Ozanimod | Macular oedema | Posterior / retinal-macular-choroidal disorders | 27 | 14.83 (10.17-21.62) | 14.78 | 1.74 | High |
| Etrasimod | Macular oedema | Posterior / retinal-macular-choroidal disorders | 11 | 33.79 (18.84-60.61) | 33.52 | 0.18 | Moderate |
| Fingolimod | Retinal oedema | Posterior / retinal-macular-choroidal disorders | 66 | 9.71 (7.60-12.42) | 9.70 | 2.11 | High |
| Fingolimod | Cystoid macular oedema | Posterior / retinal-macular-choroidal disorders | 64 | 8.52 (6.64-10.93) | 8.51 | 1.97 | High |
| Fingolimod | Retinal disorder | Posterior / retinal-macular-choroidal disorders | 53 | 5.84 (4.45-7.66) | 5.84 | 1.51 | High |
| Fingolimod | Maculopathy | Posterior / retinal-macular-choroidal disorders | 58 | 4.36 (3.37-5.65) | 4.36 | 1.25 | High |
| Fingolimod | Chorioretinopathy | Posterior / retinal-macular-choroidal disorders | 24 | 4.65 (3.12-6.94) | 4.65 | 0.85 | High |
| Fingolimod | Retinal haemorrhage | Posterior / retinal-macular-choroidal disorders | 61 | 2.53 (1.96-3.25) | 2.53 | 0.65 | High |
| Fingolimod | Scotoma | Non-specific ocular / visual symptom reports | 15 | 12.08 (7.25-20.13) | 12.08 | 0.98 | Moderate |
| Fingolimod | Diplopia | Non-specific ocular / visual symptom reports | 567 | 6.30 (5.80-6.85) | 6.27 | 2.30 | Moderate |
| Siponimod | Diplopia | Non-specific ocular / visual symptom reports | 42 | 4.89 (3.61-6.61) | 4.87 | 1.23 | Moderate |
| Fingolimod | Visual impairment | Non-specific ocular / visual symptom reports | 2,502 | 5.23 (5.02-5.44) | 5.10 | 2.19 | Moderate |
| Siponimod | Visual impairment | Non-specific ocular / visual symptom reports | 245 | 4.82 (4.24-5.47) | 4.70 | 1.80 | Moderate |
| Ozanimod | Visual impairment | Non-specific ocular / visual symptom reports | 104 | 2.03 (1.68-2.47) | 2.02 | 0.53 | Moderate |
| Fingolimod | Vision blurred | Non-specific ocular / visual symptom reports | 2,444 | 5.07 (4.87-5.29) | 4.95 | 2.14 | Moderate |
| Siponimod | Vision blurred | Non-specific ocular / visual symptom reports | 208 | 4.53 (3.94-5.19) | 4.44 | 1.69 | Moderate |
| Ozanimod | Vision blurred | Non-specific ocular / visual symptom reports | 93 | 2.03 (1.65-2.49) | 2.01 | 0.50 | Low |
| Etrasimod | Vision blurred | Non-specific ocular / visual symptom reports | 34 | 4.08 (2.91-5.72) | 4.00 | 0.92 | Moderate |
| Fingolimod | Vitreous floaters | Non-specific ocular / visual symptom reports | 164 | 4.39 (3.76-5.12) | 4.38 | 1.60 | Moderate |
| Siponimod | Vitreous floaters | Non-specific ocular / visual symptom reports | 16 | 4.03 (2.49-6.54) | 4.03 | 0.45 | Moderate |
| Fingolimod | Visual acuity reduced | Non-specific ocular / visual symptom reports | 513 | 4.64 (4.25-5.07) | 4.62 | 1.89 | Moderate |
| Fingolimod | Photopsia | Non-specific ocular / visual symptom reports | 91 | 4.07 (3.31-5.01) | 4.07 | 1.34 | Moderate |
| Siponimod | Photopsia | Non-specific ocular / visual symptom reports | 13 | 6.90 (4.04-11.79) | 6.89 | 0.62 | Moderate |
| Fingolimod | Ocular discomfort | Non-specific ocular / visual symptom reports | 158 | 3.65 (3.12-4.27) | 3.65 | 1.37 | High |
| Siponimod | Ocular discomfort | Non-specific ocular / visual symptom reports | 28 | 5.01 (3.46-7.24) | 4.99 | 1.02 | High |
| Fingolimod | Photophobia | Non-specific ocular / visual symptom reports | 273 | 3.96 (3.51-4.46) | 3.95 | 1.58 | Moderate |
| Siponimod | Photophobia | Non-specific ocular / visual symptom reports | 18 | 2.57 (1.63-4.06) | 2.57 | 0.12 | Moderate |
| Fingolimod | Eye pain | Non-specific ocular / visual symptom reports | 819 | 3.90 (3.64-4.18) | 3.87 | 1.72 | High |
| Siponimod | Eye pain | Non-specific ocular / visual symptom reports | 60 | 2.62 (2.04-3.38) | 2.61 | 0.69 | High |
| Fingolimod | Blindness transient | Non-specific ocular / visual symptom reports | 101 | 3.93 (3.23-4.78) | 3.92 | 1.33 | Moderate |
| Fingolimod | Eye disorder | Non-specific ocular / visual symptom reports | 442 | 3.61 (3.28-3.97) | 3.59 | 1.54 | Moderate |
| Siponimod | Eye disorder | Non-specific ocular / visual symptom reports | 40 | 3.19 (2.34-4.34) | 3.18 | 0.76 | Moderate |
| Fingolimod | Blindness unilateral | Non-specific ocular / visual symptom reports | 149 | 2.99 (2.55-3.52) | 2.99 | 1.11 | Moderate |
| Fingolimod | Blindness | Non-specific ocular / visual symptom reports | 422 | 2.81 (2.55-3.10) | 2.80 | 1.21 | Moderate |

Rows follow the Preferred Term order used in Supplementary Table S4; within each Preferred Term, drugs are ordered by FDA approval date. No ponesimod Preferred Term met the primary signal criteria. ROR, reporting odds ratio; CI, confidence interval; PRR, proportional reporting ratio; IC025, lower 95% bound for the information component.
