# Supplementary material for: Ocular safety signals of sphingosine-1-phosphate receptor modulators in postmarketing surveillance
Source: Front Pharmacol. 2026 Sep 25;17:1908773. doi: 10.3389/fphar.2026.1908773 (PMC13649686; doi:10.3389/fphar.2026.1908773)
Supplement: Supplementary file 6 [file Table4.docx]

**Supplementary Table S4. Class-level primary ocular reporting signals.**

| **Preferred Term** | **Presentation group** | **Reports, n** | **ROR (95% CI)** | **PRR** | **IC025** | **Robustness** |
| --- | --- | --- | --- | --- | --- | --- |
| Macular oedema | Posterior / retinal-macular-choroidal disorders | 947 | 48.18 (44.84-51.76) | 47.73 | 4.68 | High |
| Retinal oedema | Posterior / retinal-macular-choroidal disorders | 76 | 9.18 (7.30-11.55) | 9.18 | 2.12 | High |
| Cystoid macular oedema | Posterior / retinal-macular-choroidal disorders | 72 | 7.86 (6.21-9.94) | 7.85 | 1.95 | High |
| Retinal disorder | Posterior / retinal-macular-choroidal disorders | 57 | 5.14 (3.95-6.68) | 5.13 | 1.41 | High |
| Maculopathy | Posterior / retinal-macular-choroidal disorders | 65 | 4.00 (3.13-5.11) | 4.00 | 1.20 | High |
| Chorioretinopathy | Posterior / retinal-macular-choroidal disorders | 24 | 3.80 (2.55-5.67) | 3.80 | 0.66 | High |
| Retinal haemorrhage | Posterior / retinal-macular-choroidal disorders | 72 | 2.44 (1.93-3.07) | 2.44 | 0.66 | High |
| Scotoma | Non-specific ocular / visual symptom reports | 15 | 9.86 (5.92-16.44) | 9.86 | 0.92 | Moderate |
| Diplopia | Non-specific ocular / visual symptom reports | 626 | 5.69 (5.26-6.17) | 5.67 | 2.18 | Moderate |
| Visual impairment | Non-specific ocular / visual symptom reports | 2,868 | 4.90 (4.72-5.09) | 4.79 | 2.10 | Moderate |
| Vision blurred | Non-specific ocular / visual symptom reports | 2,784 | 4.72 (4.55-4.91) | 4.62 | 2.05 | Moderate |
| Vitreous floaters | Non-specific ocular / visual symptom reports | 189 | 4.14 (3.58-4.78) | 4.13 | 1.56 | Moderate |
| Visual acuity reduced | Non-specific ocular / visual symptom reports | 529 | 3.91 (3.58-4.26) | 3.89 | 1.67 | Moderate |
| Photopsia | Non-specific ocular / visual symptom reports | 106 | 3.88 (3.20-4.70) | 3.88 | 1.33 | Moderate |
| Ocular discomfort | Non-specific ocular / visual symptom reports | 192 | 3.64 (3.15-4.19) | 3.63 | 1.40 | High |
| Photophobia | Non-specific ocular / visual symptom reports | 306 | 3.63 (3.24-4.06) | 3.62 | 1.49 | Moderate |
| Eye pain | Non-specific ocular / visual symptom reports | 908 | 3.54 (3.31-3.78) | 3.51 | 1.60 | High |
| Blindness transient | Non-specific ocular / visual symptom reports | 108 | 3.43 (2.84-4.15) | 3.43 | 1.19 | Moderate |
| Eye disorder | Non-specific ocular / visual symptom reports | 508 | 3.39 (3.11-3.71) | 3.38 | 1.48 | Moderate |
| Blindness unilateral | Non-specific ocular / visual symptom reports | 163 | 2.67 (2.29-3.12) | 2.67 | 0.98 | Moderate |
| Blindness | Non-specific ocular / visual symptom reports | 474 | 2.58 (2.36-2.83) | 2.57 | 1.11 | Moderate |

Class-level High robustness required support in the 34-PT, PS+SS, and full-period-comparator analyses with n ≥10; Moderate required PS+SS and full-period-comparator support with n ≥5; remaining primary signals were Low. High was not assigned to scotoma, diplopia, visual impairment, vision blurred, visual acuity reduced, photopsia, blindness transient, eye disorder, blindness unilateral, or blindness; these PTs were classified as Moderate when the other High criteria were met. Presentation groups were used for clinical interpretation.
