# Supplementary material for: Ocular safety signals of sphingosine-1-phosphate receptor modulators in postmarketing surveillance
Source: Front Pharmacol. 2026 Sep 25;17:1908773. doi: 10.3389/fphar.2026.1908773 (PMC13649686; doi:10.3389/fphar.2026.1908773)
Supplement: Supplementary file 8 [file Table6.docx]

**Supplementary Table S6. Sensitivity analyses for drugs with low report volume.**

**Panel A. Low-report-volume sensitivity analyses**

| **Drug** | **Analysis** | **Tested drug-PT pairs, n** | **Primary signals, n** | **Primary signals with 3–5 target-event reports, n** | **Highest ROR among primary signals** |
| --- | --- | --- | --- | --- | --- |
| Ponesimod | Main analysis | 52 | 0 | 0 | NA |
| Ponesimod | 34-PT sensitivity analysis | 34 | 0 | 0 | NA |
| Ponesimod | PS+SS exposure analysis | 52 | 0 | 0 | NA |
| Ponesimod | Full-period comparator | 52 | 0 | 0 | NA |
| Ponesimod | Broad exploratory PT analysis | 781 | 0 | 0 | NA |
| Ponesimod | Neuro-ophthalmic exploratory analysis | 57 | 0 | 0 | NA |
| Etrasimod | Main analysis | 52 | 2 | 0 | 33.79 |
| Etrasimod | 34-PT sensitivity analysis | 34 | 2 | 0 | 33.79 |
| Etrasimod | PS+SS exposure analysis | 52 | 2 | 0 | 33.01 |
| Etrasimod | Full-period comparator | 52 | 2 | 0 | 32.00 |
| Etrasimod | Broad exploratory PT analysis | 781 | 2 | 0 | 33.79 |
| Etrasimod | Neuro-ophthalmic exploratory analysis | 57 | 0 | 0 | NA |

**Panel B. Restricted empirical-Bayes sensitivity for selected low-report drug-PT pairs**

| **Drug** | **Preferred Term** | **Observed, n** | **Expected, n** | **ROR (95% CI)** | **EBGM (EB05-EB95)** |
| --- | --- | --- | --- | --- | --- |
| Ponesimod | Macular oedema | 1 | 0.070 | 21.49 (4.32-107.00) | 2.43 (0.46-14.07) |
| Ponesimod | Retinal oedema | 1 | 0.030 | 50.93 (10.22-253.80) | 2.95 (0.51-18.22) |
| Ponesimod | Vision blurred | 5 | 1.741 | 3.19 (1.37-7.41) | 2.14 (1.00-4.28) |
| Etrasimod | Macular oedema | 11 | 0.347 | 33.79 (18.84-60.61) | 22.43 (13.43-35.66) |
| Etrasimod | Vision blurred | 34 | 8.625 | 4.08 (2.91-5.72) | 3.70 (2.75-4.91) |

PT, Preferred Term; ROR, reporting odds ratio; CI, confidence interval; PS, primary suspect; SS, secondary suspect; EBGM, empirical Bayes geometric mean; EB05 and EB95, posterior 5th and 95th percentiles; NA, not applicable. The main primary-suspect cohorts contained 299 reports and 12 ocular reports for ponesimod and 1,356 reports and 73 ocular reports for etrasimod. The empirical-Bayes model was fitted across all 3,905 broad exploratory drug-PT cells; Panel B presents selected ponesimod and etrasimod ocular pairs.
