# Supplementary material for: Ocular safety signals of sphingosine-1-phosphate receptor modulators in postmarketing surveillance
Source: Front Pharmacol. 2026 Sep 25;17:1908773. doi: 10.3389/fphar.2026.1908773 (PMC13649686; doi:10.3389/fphar.2026.1908773)
Supplement: Supplementary file 9 [file Table7.docx]

**Supplementary Table S7. Report-level macular/retinal oedema phenotype aggregation sensitivity.**

| **Target exposure** | **a: Target reports with phenotype** | **b: Target reports without phenotype** | **c: Comparator reports with phenotype** | **d: Comparator reports without phenotype** | **ROR (95% CI)** | **PRR** | **IC025** | **Sparse (a < 20)** |
| --- | --- | --- | --- | --- | --- | --- | --- | --- |
| S1P class | 1,066 | 97,777 | 6,735 | 18,198,525 | 29.47 (27.62–31.45) | 29.16 | 4.22 | No |
| Fingolimod | 950 | 79,845 | 6,851 | 18,216,457 | 31.65 (29.56–33.88) | 31.29 | 4.30 | No |
| Siponimod | 71 | 8,192 | 4,040 | 10,119,553 | 21.86 (17.28–27.64) | 21.68 | 2.83 | No |
| Ozanimod | 32 | 8,098 | 3,577 | 8,682,589 | 9.74 (6.89–13.77) | 9.70 | 1.65 | No |
| Ponesimod | 2 | 297 | 3,093 | 7,310,743 | 19.86 (5.72–68.99) | 19.70 | −3.83 | Yes |
| Etrasimod | 11 | 1,345 | 1,656 | 3,449,042 | 17.80 (9.94–31.86) | 17.65 | 0.54 | Yes |

Note: The macular/retinal oedema phenotype comprised macular oedema, cystoid macular oedema, and retinal oedema. After CASEID-level deduplication, each retained report was counted once within the phenotype, including reports in which more than one member PT was recorded. In each 2 × 2 table, a denotes target reports with the phenotype, b target reports without the phenotype, c comparator reports with the phenotype, and d comparator reports without the phenotype. ROR, PRR, and IC025 were calculated using the same 0.5 Haldane-Anscombe correction as the PT-level analyses. Estimates with fewer than 20 target phenotype reports (a < 20) were classified as sparse. The phenotype-level analysis was a sensitivity analysis; the PT-level analysis remained primary. PT, Preferred Term; ROR, reporting odds ratio; CI, confidence interval; PRR, proportional reporting ratio; IC025, lower 95% bound for the information component.
