# Supplementary material for: Ocular safety signals of sphingosine-1-phosphate receptor modulators in postmarketing surveillance
Source: Front Pharmacol. 2026 Sep 25;17:1908773. doi: 10.3389/fphar.2026.1908773 (PMC13649686; doi:10.3389/fphar.2026.1908773)
Supplement: Supplementary file 10 [file Table8.docx]

**Supplementary Table S8. Indication-restricted active-comparator and temporal sensitivity analyses for the report-level macular/retinal oedema phenotype.**

**Panel A. Active-comparator pools**

| **Indication** | **Comparator pool** | **Comparator drugs, n** | **Comparator drugs** | **Therapeutic class(es)** |
| --- | --- | --- | --- | --- |
| MS | Oral non-S1P MS DMT | 4 | dimethyl fumarate; diroximel fumarate; monomethyl fumarate; teriflunomide | oral fumarate; oral pyrimidine synthesis inhibitor |
| MS | Platform injectable MS DMT | 3 | glatiramer acetate; interferon beta 1a; interferon beta 1b | platform injectable |
| MS | High-efficacy non-S1P MS DMT | 6 | alemtuzumab; cladribine; natalizumab; ocrelizumab; ofatumumab; ublituximab | anti-CD20; immune reconstitution; integrin inhibitor |
| MS | Anti-CD20 MS DMT | 3 | ocrelizumab; ofatumumab; ublituximab | anti-CD20 |
| MS | All non-S1P MS DMT | 13 | alemtuzumab; cladribine; dimethyl fumarate; diroximel fumarate; glatiramer acetate; interferon beta 1a; interferon beta 1b; monomethyl fumarate; natalizumab; ocrelizumab; ofatumumab; teriflunomide; ublituximab | anti-CD20; immune reconstitution; integrin inhibitor; oral fumarate; oral pyrimidine synthesis inhibitor; platform injectable |
| UC/IBD | Biologic non-S1P UC/IBD therapy | 7 | adalimumab; golimumab; infliximab; mirikizumab; risankizumab; ustekinumab; vedolizumab | anti-TNF; IL-12/23 inhibitor; IL-23 inhibitor; integrin inhibitor |
| UC/IBD | JAK inhibitor UC/IBD therapy | 2 | tofacitinib; upadacitinib | JAK inhibitor |
| UC/IBD | All non-S1P UC/IBD therapy | 9 | adalimumab; golimumab; infliximab; mirikizumab; risankizumab; tofacitinib; upadacitinib; ustekinumab; vedolizumab | anti-TNF; IL-12/23 inhibitor; IL-23 inhibitor; integrin inhibitor; JAK inhibitor |

**Panel B. MS class-level active-comparator analyses**

| **Comparison** | **Target reports, n** | **Comparator reports, n** | **Target phenotype reports, n** | **Comparator phenotype reports, n** | **ROR (95% CI)** | **Interpretation** |
| --- | --- | --- | --- | --- | --- | --- |
| S1P class vs all non-S1P MS DMTs | 67,185 | 447,862 | 693 | 106 | 43.85 (35.75-53.78) | Principal non-sparse |
| S1P class vs oral non-S1P MS DMTs | 67,185 | 140,609 | 693 | 33 | 43.77 (30.94-61.91) | Principal non-sparse |
| S1P class vs platform injectable MS DMTs | 67,185 | 134,407 | 693 | 43 | 32.22 (23.71-43.77) | Principal non-sparse |
| S1P class vs high-efficacy non-S1P MS DMTs | 67,185 | 172,846 | 693 | 30 | 59.10 (41.12-84.94) | Principal non-sparse |
| S1P class vs anti-CD20 MS DMTs | 67,185 | 58,130 | 693 | 12 | 48.49 (27.71-84.85) | Supportive sparse |

**Panel C. MS individual-agent active-comparator analyses**

| **Comparison** | **Target reports, n** | **Comparator reports, n** | **Target phenotype reports, n** | **Comparator phenotype reports, n** | **ROR (95% CI)** | **Interpretation** |
| --- | --- | --- | --- | --- | --- | --- |
| Fingolimod vs all non-S1P MS DMTs | 57,073 | 447,862 | 627 | 106 | 46.74 (38.05-57.41) | Principal non-sparse |
| Fingolimod vs oral non-S1P MS DMTs | 57,073 | 140,609 | 627 | 33 | 46.65 (32.95-66.05) | Principal non-sparse |
| Fingolimod vs platform injectable MS DMTs | 57,073 | 134,407 | 627 | 43 | 34.34 (25.25-46.70) | Principal non-sparse |
| Fingolimod vs high-efficacy non-S1P MS DMTs | 57,073 | 172,846 | 627 | 30 | 62.99 (43.79-90.60) | Principal non-sparse |
| Fingolimod vs anti-CD20 MS DMTs | 57,073 | 58,130 | 627 | 12 | 51.69 (29.52-90.49) | Supportive sparse |
| Siponimod vs all non-S1P MS DMTs | 5,347 | 187,643 | 56 | 45 | 44.02 (29.77-65.11) | Principal non-sparse |
| Siponimod vs oral non-S1P MS DMTs | 5,347 | 57,814 | 56 | 15 | 39.82 (22.68-69.89) | Supportive sparse |
| Siponimod vs platform injectable MS DMTs | 5,347 | 39,111 | 56 | 11 | 36.30 (19.24-68.49) | Supportive sparse |
| Siponimod vs high-efficacy non-S1P MS DMTs | 5,347 | 90,718 | 56 | 19 | 49.66 (29.66-83.16) | Supportive sparse |
| Siponimod vs anti-CD20 MS DMTs | 5,347 | 55,149 | 56 | 12 | 47.10 (25.51-86.97) | Supportive sparse |
| Ozanimod vs all non-S1P MS DMTs | 4,602 | 156,932 | 9 | 37 | 8.65 (4.24-17.64) | Supportive sparse |
| Ozanimod vs oral non-S1P MS DMTs | 4,602 | 46,505 | 9 | 12 | 7.69 (3.31-17.89) | Supportive sparse |
| Ozanimod vs platform injectable MS DMTs | 4,602 | 29,438 | 9 | 9 | 6.41 (2.61-15.76) | Supportive sparse |
| Ozanimod vs high-efficacy non-S1P MS DMTs | 4,602 | 80,989 | 9 | 16 | 10.15 (4.57-22.56) | Supportive sparse |
| Ozanimod vs anti-CD20 MS DMTs | 4,602 | 52,002 | 9 | 12 | 8.60 (3.70-20.01) | Supportive sparse |
| Ponesimod vs all non-S1P MS DMTs | 163 | 128,874 | 1 | 31 | 37.76 (7.29-195.66) | Feasibility only; minimum criteria not met |
| Ponesimod vs oral non-S1P MS DMTs | 163 | 35,081 | 1 | 9 | 34.08 (6.05-192.02) | Feasibility only; minimum criteria not met |
| Ponesimod vs platform injectable MS DMTs | 163 | 21,899 | 1 | 6 | 31.09 (5.23-184.76) | Feasibility only; minimum criteria not met |
| Ponesimod vs high-efficacy non-S1P MS DMTs | 163 | 71,894 | 1 | 16 | 40.21 (7.51-215.45) | Feasibility only; minimum criteria not met |
| Ponesimod vs anti-CD20 MS DMTs | 163 | 48,531 | 1 | 12 | 35.83 (6.54-196.25) | Feasibility only; minimum criteria not met |

**Panel D. UC/IBD exploratory active-comparator analyses**

| **Comparison** | **Target reports, n** | **Comparator reports, n** | **Target phenotype reports, n** | **Comparator phenotype reports, n** | **ROR (95% CI)** | **Interpretation** |
| --- | --- | --- | --- | --- | --- | --- |
| S1P class vs all non-S1P UC/IBD therapies | 2,293 | 76,959 | 22 | 10 | 72.59 (34.85-151.19) | Exploratory sparse |
| S1P class vs biologic non-S1P UC/IBD therapies | 2,293 | 67,524 | 22 | 7 | 89.17 (38.98-203.98) | Exploratory sparse |
| S1P class vs JAK inhibitor non-S1P UC/IBD therapies | 2,293 | 9,435 | 22 | 3 | 26.69 (8.65-82.40) | Exploratory sparse |
| Ozanimod vs all non-S1P UC/IBD therapies | 1,793 | 76,959 | 18 | 10 | 76.36 (35.76-163.08) | Exploratory sparse |
| Ozanimod vs biologic non-S1P UC/IBD therapies | 1,793 | 67,524 | 18 | 7 | 93.80 (40.10-219.41) | Exploratory sparse |
| Ozanimod vs JAK inhibitor non-S1P UC/IBD therapies | 1,793 | 9,435 | 18 | 3 | 28.08 (8.95-88.12) | Exploratory sparse |
| Etrasimod vs all non-S1P UC/IBD therapies | 500 | 36,170 | 4 | 8 | 38.56 (12.26-121.30) | Exploratory sparse |
| Etrasimod vs biologic non-S1P UC/IBD therapies | 500 | 31,093 | 4 | 5 | 51.23 (14.69-178.64) | Exploratory sparse |
| Etrasimod vs JAK inhibitor non-S1P UC/IBD therapies | 500 | 5,077 | 4 | 3 | 13.14 (3.24-53.28) | Exploratory sparse |

**Panel E. Fingolimod temporal sensitivity versus all non-S1P MS DMTs**

| **Period or method** | **Target reports, n** | **Comparator reports, n** | **Target phenotype reports, n** | **Comparator phenotype reports, n** | **ROR or common OR (95% CI)** |
| --- | --- | --- | --- | --- | --- |
| Full approval-aligned period | 57,073 | 447,862 | 627 | 106 | 46.74 (38.05-57.41) |
| First 2 years after approval | 601 | 2,300 | 16 | 0 | 129.66 (7.77-2164.47) |
| Years 2 to 5 after approval | 9,626 | 143,121 | 146 | 31 | 70.20 (47.74-103.21) |
| After the first 2 years | 56,472 | 445,562 | 611 | 106 | 45.79 (37.26-56.26) |
| After the first 5 years | 46,846 | 302,441 | 465 | 75 | 40.19 (31.51-51.27) |
| Calendar-quarter-stratified common OR | 57,073 | 447,862 | 627 | 106 | 44.01 (35.20-55.04) |

Active-comparator analyses used the report-level macular/retinal oedema phenotype. Minimum analysis criteria were at least 100 target reports, 300 comparator reports, three target phenotype reports, and three comparator phenotype reports. Comparisons with either phenotype count below 20 were classified as sparse. Non-sparse MS comparisons were interpreted as principal sensitivity results, sparse MS comparisons as supportive, and UC/IBD comparisons as exploratory; ponesimod comparisons below the minimum event-count criterion are shown for feasibility. Detailed eligibility and co-exposure exclusions are described in the Supplementary Methods. S1P, sphingosine-1-phosphate; MS, multiple sclerosis; UC, ulcerative colitis; IBD, inflammatory bowel disease; DMT, disease-modifying therapy; ROR, reporting odds ratio; OR, odds ratio; CI, confidence interval.
