# Supplementary material for: Ocular safety signals of sphingosine-1-phosphate receptor modulators in postmarketing surveillance
Source: Front Pharmacol. 2026 Sep 25;17:1908773. doi: 10.3389/fphar.2026.1908773 (PMC13649686; doi:10.3389/fphar.2026.1908773)
Supplement: Supplementary file 11 [file Table9.docx]

**Supplementary Table S9. Targeted Canada Vigilance external validation of posterior/retinal-macular-choroidal FAERS findings.**

**Panel A. Report-level macular/retinal oedema phenotype**

| **Analysis level** | **Canadian market-entry date** | **Target Suspect reports, n** | **Comparator reports, n** | **Target phenotype reports, n** | **Comparator phenotype reports, n** | **ROR (95% CI)** | **PRR** | **IC025** | **Analysis status** |
| --- | --- | --- | --- | --- | --- | --- | --- | --- | --- |
| S1P class | 2011-03-28 | 4,683 | 904,557 | 109 | 292 | 74.00 (59.30-92.35) | 72.29 | 3.74 | Non-sparse |
| Fingolimod | 2011-03-28 | 3,661 | 905,579 | 93 | 308 | 76.89 (60.86-97.13) | 74.95 | 3.61 | Non-sparse |
| Siponimod | 2020-04-24 | 693 | 426,438 | 16 | 168 | 61.61 (36.95-102.72) | 60.17 | 0.57 | Sparse |
| Ozanimod | 2020-11-12 | 108 | 388,806 | 1 | 170 | 31.81 (6.30-160.47) | 31.38 | -8.12 | Feasibility only |
| Ponesimod | 2021-11-01 | 0 | 319,373 | 0 | 131 | NE | NE | NE | Not estimable |
| Etrasimod | 2024-04-18 | 238 | 140,803 | 0 | 48 | 6.08 (0.37-98.96) | 6.07 | -8.01 | Feasibility only |

**Panel B. Secondary targeted PT-level external validation**

| **Preferred Term** | **Analysis level** | **Target Suspect reports, n** | **Comparator reports, n** | **Target PT reports, n** | **Comparator PT reports, n** | **ROR (95% CI)** | **PRR** | **IC025** | **BH q value** | **Sparse-data status** |
| --- | --- | --- | --- | --- | --- | --- | --- | --- | --- | --- |
| Macular oedema | S1P class | 4,683 | 904,557 | 104 | 170 | 121.04 (94.72-154.68) | 118.36 | 3.81 | <0.001 | Non-sparse |
| Macular oedema | Fingolimod | 3,661 | 905,579 | 89 | 185 | 122.28 (94.81-157.70) | 119.31 | 3.63 | <0.001 | Non-sparse |
| Macular oedema | Siponimod | 693 | 426,438 | 15 | 100 | 96.91 (56.47-166.32) | 94.77 | -0.19 | <0.001 | Sparse |
| Macular oedema | Ozanimod | 108 | 388,806 | 1 | 104 | 51.90 (10.24-263.01) | 51.20 | -10.91 | <0.001 | Feasibility only |
| Macular oedema | Ponesimod | 0 | 319,373 | 0 | 77 | NE | NE | NE | NE | Not estimable |
| Macular oedema | Etrasimod | 238 | 140,803 | 0 | 23 | 12.56 (0.76-207.37) | 12.53 | -10.96 | 1.000 | Feasibility only |
| Retinal oedema | S1P class | 4,683 | 904,557 | 3 | 91 | 7.39 (2.54-21.50) | 7.39 | -1.48 | <0.001 | Sparse |
| Retinal oedema | Fingolimod | 3,661 | 905,579 | 2 | 92 | 6.69 (1.90-23.50) | 6.68 | -2.20 | 0.019 | Feasibility only |
| Retinal oedema | Siponimod | 693 | 426,438 | 1 | 51 | 17.93 (3.53-91.09) | 17.90 | -5.79 | 0.004 | Feasibility only |
| Retinal oedema | Ozanimod | 108 | 388,806 | 0 | 49 | 36.19 (2.22-590.53) | 36.03 | -19.06 | 1.000 | Feasibility only |
| Retinal oedema | Ponesimod | 0 | 319,373 | 0 | 40 | NE | NE | NE | NE | Not estimable |
| Retinal oedema | Etrasimod | 238 | 140,803 | 0 | 19 | 15.14 (0.91-251.41) | 15.11 | -11.99 | 1.000 | Feasibility only |
| Cystoid macular oedema | S1P class | 4,683 | 904,557 | 2 | 39 | 12.23 (3.40-43.92) | 12.22 | -2.81 | <0.001 | Feasibility only |
| Cystoid macular oedema | Fingolimod | 3,661 | 905,579 | 2 | 39 | 15.66 (4.36-56.25) | 15.65 | -3.22 | <0.001 | Feasibility only |
| Cystoid macular oedema | Siponimod | 693 | 426,438 | 0 | 21 | 14.30 (0.87-236.30) | 14.29 | -11.68 | 1.000 | Feasibility only |
| Cystoid macular oedema | Ozanimod | 108 | 388,806 | 0 | 20 | 87.40 (5.25-1454.22) | 87.00 | -30.67 | 1.000 | Feasibility only |
| Cystoid macular oedema | Ponesimod | 0 | 319,373 | 0 | 16 | NE | NE | NE | NE | Not estimable |
| Cystoid macular oedema | Etrasimod | 238 | 140,803 | 0 | 8 | 34.73 (2.00-603.37) | 34.66 | -18.21 | 1.000 | Feasibility only |
| Retinal disorder | S1P class | 4,683 | 904,557 | 9 | 132 | 13.87 (7.18-26.81) | 13.85 | 0.27 | <0.001 | Sparse |
| Retinal disorder | Fingolimod | 3,661 | 905,579 | 7 | 134 | 13.82 (6.62-28.84) | 13.79 | -0.17 | <0.001 | Sparse |
| Retinal disorder | Siponimod | 693 | 426,438 | 2 | 43 | 35.44 (9.88-127.07) | 35.31 | -5.43 | <0.001 | Feasibility only |
| Retinal disorder | Ozanimod | 108 | 388,806 | 0 | 40 | 44.24 (2.70-724.00) | 44.04 | -21.24 | 1.000 | Feasibility only |
| Retinal disorder | Ponesimod | 0 | 319,373 | 0 | 38 | NE | NE | NE | NE | Not estimable |
| Retinal disorder | Etrasimod | 238 | 140,803 | 0 | 19 | 15.14 (0.91-251.41) | 15.11 | -11.99 | 1.000 | Feasibility only |
| Maculopathy | S1P class | 4,683 | 904,557 | 6 | 63 | 19.79 (8.83-44.39) | 19.77 | -0.67 | <0.001 | Sparse |
| Maculopathy | Fingolimod | 3,661 | 905,579 | 5 | 64 | 21.12 (8.84-50.47) | 21.09 | -1.17 | <0.001 | Sparse |
| Maculopathy | Siponimod | 693 | 426,438 | 1 | 31 | 29.32 (5.69-151.11) | 29.26 | -7.62 | <0.001 | Feasibility only |
| Maculopathy | Ozanimod | 108 | 388,806 | 0 | 31 | 56.88 (3.46-935.40) | 56.62 | -24.32 | 1.000 | Feasibility only |
| Maculopathy | Ponesimod | 0 | 319,373 | 0 | 27 | NE | NE | NE | NE | Not estimable |
| Maculopathy | Etrasimod | 238 | 140,803 | 0 | 7 | 39.36 (2.24-691.06) | 39.28 | -19.41 | 1.000 | Feasibility only |
| Chorioretinopathy | S1P class | 4,683 | 904,557 | 1 | 22 | 12.88 (2.47-67.26) | 12.87 | -4.77 | 0.023 | Feasibility only |
| Chorioretinopathy | Fingolimod | 3,661 | 905,579 | 1 | 22 | 16.49 (3.16-86.15) | 16.49 | -5.43 | 0.007 | Feasibility only |
| Chorioretinopathy | Siponimod | 693 | 426,438 | 0 | 6 | 47.30 (2.66-840.47) | 47.27 | -21.36 | 1.000 | Feasibility only |
| Chorioretinopathy | Ozanimod | 108 | 388,806 | 0 | 6 | 275.65 (15.43-4923.45) | 274.39 | -55.97 | 1.000 | Feasibility only |
| Chorioretinopathy | Ponesimod | 0 | 319,373 | 0 | 3 | NE | NE | NE | NE | Not estimable |
| Chorioretinopathy | Etrasimod | 238 | 140,803 | 0 | 1 | 196.79 (8.00-4843.14) | 196.38 | -41.53 | 1.000 | Feasibility only |
| Retinal haemorrhage | S1P class | 4,683 | 904,557 | 8 | 190 | 8.63 (4.34-17.17) | 8.62 | 0.10 | <0.001 | Sparse |
| Retinal haemorrhage | Fingolimod | 3,661 | 905,579 | 7 | 191 | 9.70 (4.67-20.14) | 9.69 | -0.10 | <0.001 | Sparse |
| Retinal haemorrhage | Siponimod | 693 | 426,438 | 1 | 81 | 11.33 (2.25-57.07) | 11.31 | -4.56 | 0.036 | Feasibility only |
| Retinal haemorrhage | Ozanimod | 108 | 388,806 | 0 | 80 | 22.25 (1.37-361.15) | 22.16 | -14.73 | 1.000 | Feasibility only |
| Retinal haemorrhage | Ponesimod | 0 | 319,373 | 0 | 61 | NE | NE | NE | NE | Not estimable |
| Retinal haemorrhage | Etrasimod | 238 | 140,803 | 0 | 33 | 8.81 (0.54-144.20) | 8.79 | -9.32 | 1.000 | Feasibility only |

S1P, sphingosine-1-phosphate; PT, Preferred Term; ROR, reporting odds ratio; CI, confidence interval; PRR, proportional reporting ratio; IC025, lower 95% bound for the information component; BH, Benjamini-Hochberg; NE, not estimable.

The primary phenotype comprised Macular oedema, Cystoid macular oedema, and Retinal oedema, counted once per deduplicated report. Target reports required Suspect role; comparators were all other eligible reports in the corresponding market window. The class target was the union of unique reports containing any included S1P modulator as Suspect; multi-S1P reports were counted once. Panel B evaluated separately the seven posterior/retinal-macular-choroidal PTs that met the class-level primary FAERS signal criteria; they were not combined into a broader phenotype. The family comprised 42 comparisons; Benjamini-Hochberg adjustment used m=42, with q values reported for 35 evaluable comparisons; seven ponesimod comparisons were not estimable. Sparse denotes fewer than 20 endpoint-positive target reports; Feasibility only denotes fewer than 3. Zero-event estimates used the same 0.5 continuity correction as the FAERS analyses; ponesimod was not estimable because no eligible Suspect reports were identified. A sensitivity analysis without Duplicate-link collapse yielded a similar class-level estimate (110/4,824; ROR, 70.47; 95% CI, 56.66–87.65) to the primary analysis (109/4,683; ROR, 74.00; 95% CI, 59.30–92.35).
